# A complex of α_6_ integrin and E-cadherin drives liver metastasis of colorectal cancer cells through hepatic angiopoietin-like 6

**DOI:** 10.1002/emmm.201101164

**Published:** 2012-10-16

**Authors:** Serena Marchiò, Marco Soster, Sabrina Cardaci, Andrea Muratore, Alice Bartolini, Vanessa Barone, Dario Ribero, Maria Monti, Paola Bovino, Jessica Sun, Raffaella Giavazzi, Sofia Asioli, Paola Cassoni, Lorenzo Capussotti, Piero Pucci, Antonella Bugatti, Marco Rusnati, Renata Pasqualini, Wadih Arap, Federico Bussolino

**Affiliations:** 1 Department of Oncology, University of TurinCandiolo, Italy; 2 Lab of Tumor Microenvironment, Institute for Cancer Research at Candiolo (IRCC)Candiolo, Italy; 3 APAvadis Biotechnologies srl, BioIndustry Park S. FumeroColleretto Giacosa, Italy; 4 Unit of Surgical Oncology, IRCCCandiolo, Italy; 5 Unit of Hepato-Biliary-Pancreatic and Digestive Surgery, Mauriziano HospitalTurin, Italy; 6 CEINGE Advanced Biotechnology, Department of Organic Chemistry and Biochemistry, Federico II UniversityNaples, Italy; 7 David H. Koch Center, The University of Texas MD Anderson Cancer CenterHouston, TX, USA; 8 Lab of Biology and Treatment of Metastasis, Department of Oncology, Mario Negri Institute for Pharmacological ResearchMilan, Italy; 9 Department of Biomedical Sciences and Human Oncology, University of TurinTurin, Italy; 10 Department of Medical Sciences, University of BresciaBrescia, Italy; 11 Lab of Vascular Oncology, IRCCCandiolo, Italy

**Keywords:** angiopoietin-like 6, E-cadherin, metastatic colorectal cancer, microenvironment, α_6_ integrin

## Abstract

Homing of colorectal cancer (CRC) cells to the liver is a non-random process driven by a crosstalk between tumour cells and components of the host tissue. Here we report the isolation of a liver metastasis-specific peptide ligand (CGIYRLRSC) that binds a complex of E-cadherin and α_6_ integrin on the surface of CRC cells. We identify angiopoietin-like 6 protein as a peptide-mimicked natural ligand enriched in hepatic blood vessels of CRC patients. We demonstrate that an interaction between hepatic angiopoietin-like 6 and tumoural α_6_ integrin/E-cadherin drives liver homing and colonization by CRC cells, and that CGIYRLRSC inhibits liver metastasis through interference with this ligand/receptor system. Our results indicate a mechanism for metastasis whereby a soluble factor accumulated in normal vessels functions as a specific ligand for circulating cancer cells. Consistently, we show that high amounts of coexpressed α_6_ integrin and E-cadherin in primary tumours represent a poor prognostic factor for patients with advanced CRC.

## INTRODUCTION

Up to 25% of patients diagnosed with colorectal cancer (CRC) present with liver metastases; in a further 30–40% of cases, metastases will develop within 2 years from the resection of the primary tumour (Parkin et al, 2005). Patients with operable liver-confined lesions might be cured by resection (Tomlinson et al, 2007), but surgical cures are relatively rare in this setting. Most patients with metastatic disease are candidates for systemic chemotherapy to palliate symptoms and, potentially, to downstage tumours to a resectable status (Adam et al, 2004). Without treatment, the median survival of patients with liver metastases is 6–8 months, and 5-year survival rates are <5% (Wagner et al, 1984). The introduction of targeted biodrugs, *e.g.* Bevacizumab (Hurwitz et al, 2004), Cetuximab (Jonker et al, 2007) and Panitumumab (Saltz et al, 2006), has prolonged the median survival expectancy up to 24 months.

However, a pharmacological cure is still anecdotal, and hepatic metastasis remains the central clinical challenge in the management of CRC. It is now clear that only new classes of drugs that attack new targets will substantially improve the state of the art for CRC care (Saltz, 2008). It has long been recognized that several proteins integrate their action during the natural history of metastatic CRC (Fearon & Vogelstein, 1990; Vogelstein et al, 1988); in addition to alterations in tumour cells, a pivotal contribution to metastatic onset comes from components of the host tissue and stroma (Hanahan & Weinberg, 2011). Based on these assumptions, insights into the molecular mechanisms underlying this disease have begun to emerge through genomics and proteomics (Koh et al, 2008; Nibbe et al, 2009). However, the fact that mRNA levels are not necessarily correlated with protein amounts confers limitations for gene expression analyses; alternatively, proteomics is time-consuming and expensive, features that render its routine use difficult.

We here report an alternative approach, based on screening combinatorial peptide libraries *ex vivo* on liver metastases obtained from CRC patients during surgery. This allowed selecting short peptide motifs as specific ligands for the microenvironment of human liver metastasis. We combined bioinformatics, genetics and biochemistry tools to uncover candidate proteins with potential ligand (*i.e.* peptide-like) or receptor (*i.e.* peptide-binding) properties. This approach led to the identification of angiopoietin-like 6, α_6_ integrin, and E-cadherin as key molecular interactors.

Angiopoietin-like 6 is a secreted glycoprotein; the corresponding mRNA has been detected exclusively in the liver in humans (Kim et al, 2000). Although it shares a common structure with other members of the family, angiopoietin-like 6 does not bind the Tie1 or Tie2 receptors (Oike et al, 2005). Angiopoietin-like 6 regulates angiogenesis by (i) prevention of endothelial cell apoptosis (Kim et al, 2000), (ii) induction of endothelial cell migration and vascular leakiness (Oike et al, 2004) and (iii) enhancement of blood flow (Urano et al, 2008). There is evidence that RGD-binding integrins might be involved in angiopoietin-like 6-mediated cell adhesion and migration (Zhang et al, 2006), although a direct interaction with integrins was not described.

Alpha 6 Integrin, complexed with the β_1_ or β_4_ subunit, is a receptor for laminin (Humphries et al, 2006), with a role in angiogenesis (Gonzalez et al, 2002) and cancer progression (Rabinovitz et al, 2001) through both direct and indirect mechanisms. Among these, (i) relocalization of α_6_β_4_ integrin from hemidesmosomes to the edge of lamellipodia and filopodia has been related to a functional switch from adhesion to migration (Germain et al, 2009; Mercurio et al, 2001), (ii) interaction of α_6_β_4_ integrin with tyrosine-kinase receptors has been shown to amplify pro-invasive signals (Bertotti et al, 2005; Guo et al, 2006; Kawano et al, 2010), (iii) α_6_β_1_ and α_6_β_4_ integrins mediate CRC cell binding to hepatocytes (Enns et al, 2004) and extravasation during the onset of metastasis (Robertson et al, 2009), although the molecular mechanisms remain to be elucidated.

E-cadherin is a well-described oncosuppressor whose expression in the primary tumour counteracts cell detachment and is therefore associated with a better outcome (Christofori, 2003). Decreased production of E-cadherin is one of the central events underlying epithelial–mesenchymal transition and carcinoma progression in response to cellular events such as (i) acquisition of loss-of-function mutations and loss-of-heterozygosis for the mutant allele (Ilyas et al, 1997), (ii) transcriptional or epigenetic repression (Natalwala et al, 2008) and (iii) aberrant cellular localization (Elzagheid et al, 2006). In contrast, the role of E-cadherin in late stages of cancer progression needs further characterization. Remarkably, different reports show that both mRNA and protein expression are regained in a subset of liver metastases (Wells et al, 2008), and increased levels of E-cadherin have been detected in liver metastases in comparison to metastases in other sites (Elzagheid et al, 2006).

## RESULTS

### Peptide ligands specific for the microenvironment of human liver metastases secondary to CRC display conserved amino acid motifs

**Figure 1:**
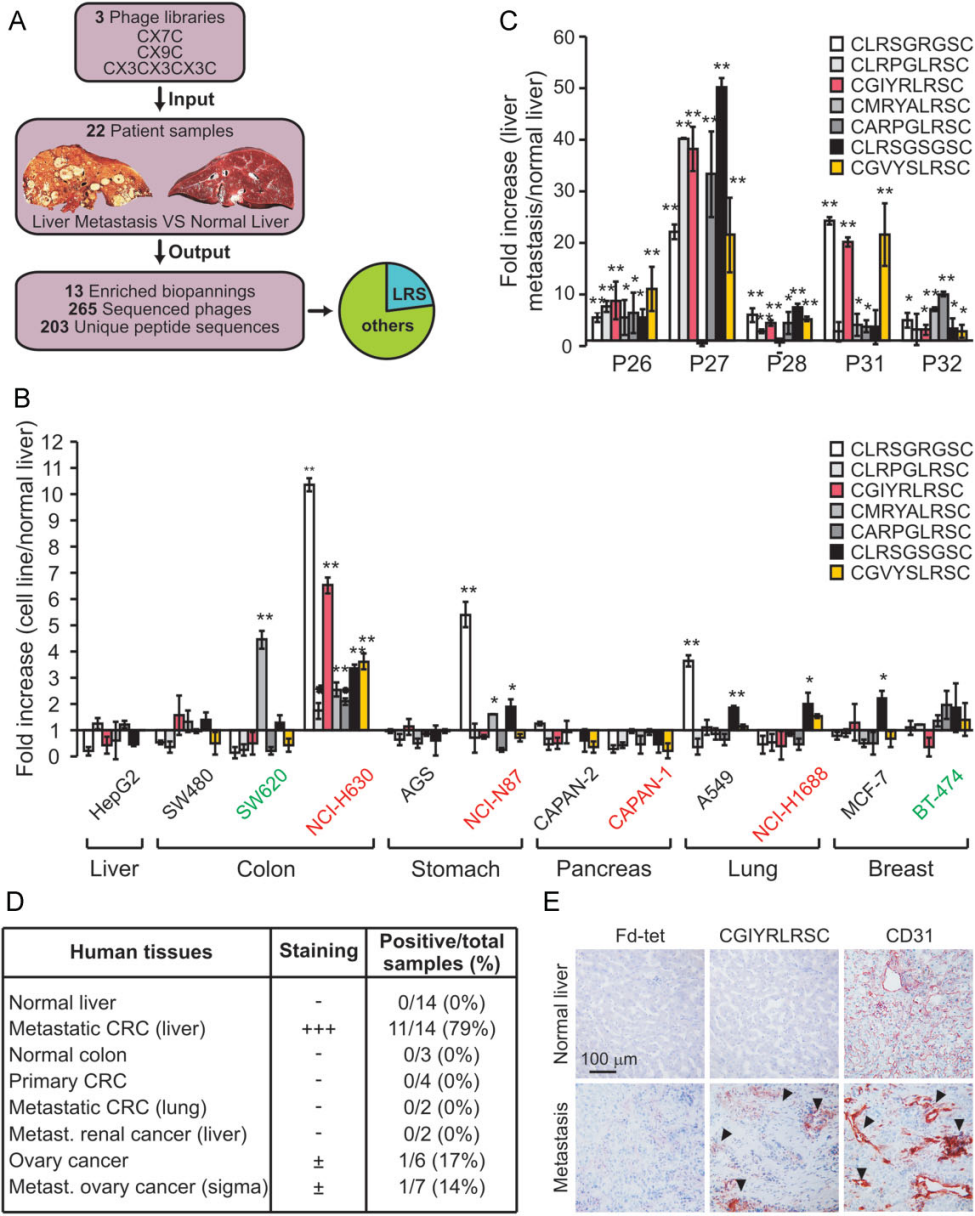
Phage display-selected peptides as ligands for human liver metastasis from CRC **A.** Three phage displayed-peptide libraries were screened on sample pairs from 22 patients, by preadsorption on control tissues (grossly normal liver) followed by enrichment on target tissues (hepatic metastasis). In 13 experiments, a selective enrichment in phage binding was observed. Sequencing of the derived 265 phage inserts revealed 203 unique metastasis-binding peptide sequences; of these, 23% shared the LRS tripeptide motif.**B–E.** CLRSGRGSC-, CLRPGLRSC-, CGIYRLRSC-, CMRYALRSC-, CARPGLRSC-, CLRSGSGSC- or CGVYSLRSC-phage was incubated with the indicated cell lines (**B**, in black: primary tumour; in red: hepatic metastasis; in green: metastasis in other sites) or with suspended cells of human liver metastasis from five patients (**C**) Numbers were normalized first to the degree of binding to the insertless fd-tet phage and subsequently to that of normal liver cells. Results are shown as mean ± standard deviation for each experimental point in five independent experiments. Statistical significance was evaluated by ANOVA followed by Bonferroni's post-test, keeping as a reference a 1.5-fold threshold assumed as positive phage binding. For phage overlay assays, 10-µm cryostatic tissue sections were incubated with 10^8^ TU of either fd-tet or CGIYRLRSC-phage (**D**). A representative assay is shown. The insertless fd-tet phage was used as a negative control; blood vessels were stained with anti-CD31 antibody, arrowheads point to the same vessels in consecutive sections (**E**). Asterisks indicate the following *p*-value ranges: *p < 0.05, **p < 0.01, ***p < 0.001.

We isolated heterogeneous cell populations by tissue fractionation of human liver metastases immediately after surgical removal, and we used cells extracted from matching, grossly normal livers as negative controls. We screened phage-displayed random peptide libraries with the general arrangements CX_7_C, CX_9_C, and CX_3_CX_3_CX_3_C (C = Cys and X = any aa) on a panel of tumour/normal paired samples in 22 independent biopanning experiments. A total of 265 phage clones were recovered, purified and DNA-sequenced (Scott & Smith, 1990; Smith & Scott, 1993). Analysis of the corresponding peptides revealed 203 unique metastasis-binding sequences. The tripeptide LRS was the most represented, being shared by ∼23% of the clones (Fig 1A).

The enrichment for specific sequences among the metastasis-binding peptides was suggestive of a role as a relevant ligand motif within the microenvironment of the liver metastasis. For an initial molecular analysis, we selected a panel of peptides (*n* = 7) derived from screenings with the CX_7_C library: CLRSGRGSC, CLRPGLRSC, CGIYRLRSC, CMRYALRSC, CARPGLRSC, CLRSGSGSC and CGVYSLRSC. We first evaluated binding of the corresponding phage clones to human cell lines from different primary tumours and metastases (*n* = 12). All these phages showed binding preference for NCI-H630 cells (liver metastasis from CRC), with CGIYRLRSC and CGVYSLRSC as the most specific ligands (Fig 1B). We next evaluated phage binding to cells freshly extracted from patients (*n* = 5) and found that most clones recognized liver metastases with high selectivity (Fig 1C). Specific binding was higher on primary cells (range, 2.8–50.2) compared to the NCI-H630 cell line (range, 1.6–10.3), a result indicating that cell types other than epithelial cells might be targeted and/or that in fresh human tissues the targets are more accessible than in the corresponding cell lines. To investigate the tissue distribution of potential targets, we performed binding overlay assays on a panel of different human epithelial tumour types and their corresponding metastases (*n* = 31). Consistently, CGIYRLRSC-phage identified ∼80% of the liver metastases from CRCs, with barely detectable staining of other tissue types (Fig 1D). Notably, cell clusters in close proximity to blood vessels were generally well targeted by the CGIYRLRSC-phage (Fig 1E). In these same assays, the CGVYSLRSC-phage showed similar staining (data not shown).

### Angiopoietin-like 6 mimics two metastasis-binding peptides and is enriched in hepatic blood vessels of patients with metastatic CRC

**Figure 2:**
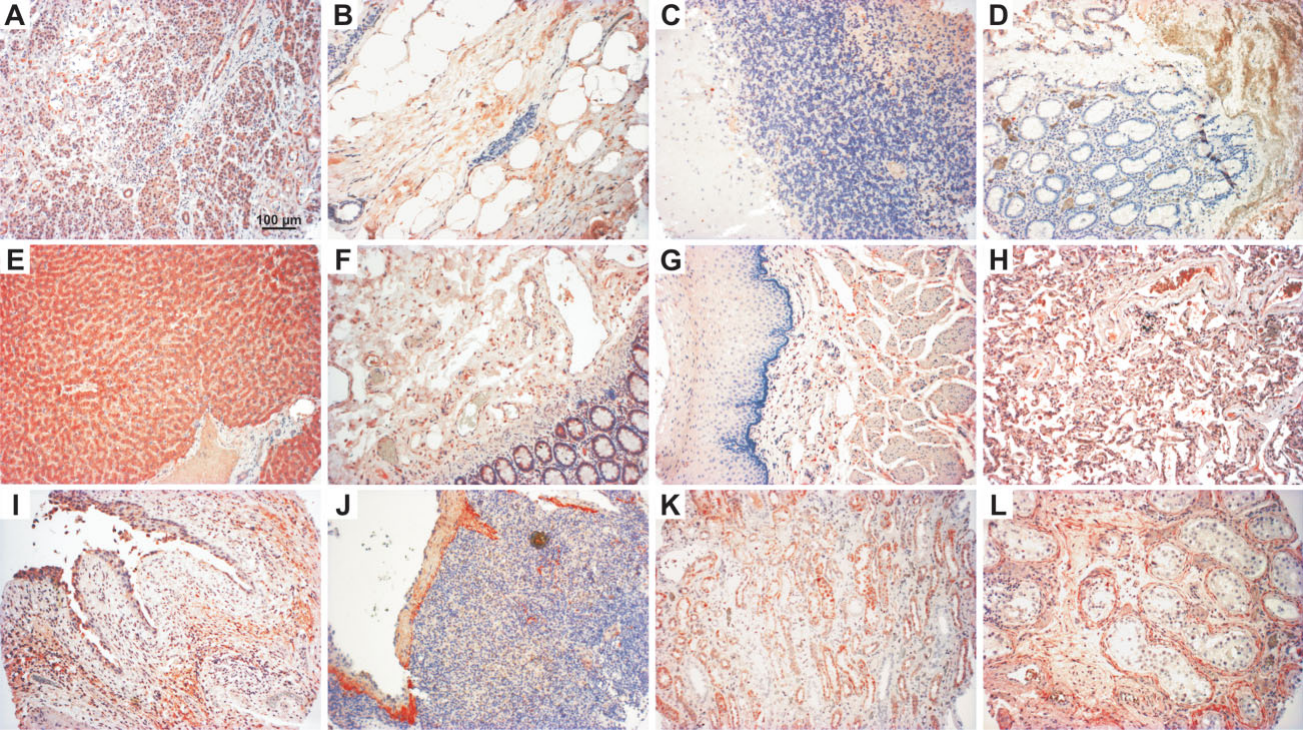
Angiopoietin-like 6 is highly expressed in hepatic tissues in humans **A–L.** Paraffin-embedded normal tissue samples from healthy donors were cut in 5-µm sections and stained with the rabbit polyclonal anti-angiopoietin-like 6 antibody. (**A**) pancreas, (**B**) breast, (**C**) cerebellum, (**D**) stomach, (**E**) liver, (**F**) intestine, (**G**) oesophagus, (**H**) lung, (**I**) bladder, (**J**) spleen, (**K**) kidney, (**L**) testis.

Because the biopanning experiments were performed on a mixture of intact cells and tissue stroma, the selected peptides represent prototype ligands for the extracellular microenvironment of liver metastases. To identify natural ligands mimicked by these peptides, we focused a BLAST query on proteins similar to the closely related peptide inserts GIYRLRS and GVYSLRS. Among the soluble factors identified (Supporting Information Table S1), angiopoietin-like 6 is of particular interest, because it shares similarity with these peptides in two regions of its fibrinogen-like domain. Interestingly, angiopoietin-like 6 mRNA has been detected exclusively in the liver in humans (Kim et al, 2000). To investigate whether angiopoietin-like 6 could actually act as a ligand for the hepatic homing of metastatic CRC cells, we first evaluated the presence of this protein in several tissue types from healthy donors. We confirmed that normal hepatic tissues produce the highest amounts of angiopoietin-like 6, although its expression was detectable in most tissues, a result possibly related to the improved sensitivity of our staining protocol compared to the whole tissue mRNA analysis of Kim et al. Remarkably, the lung, another common site of CRC metastasis, was one of the tissues with the highest angiopoietin-like 6 levels (Fig 2).

**Figure 3:**
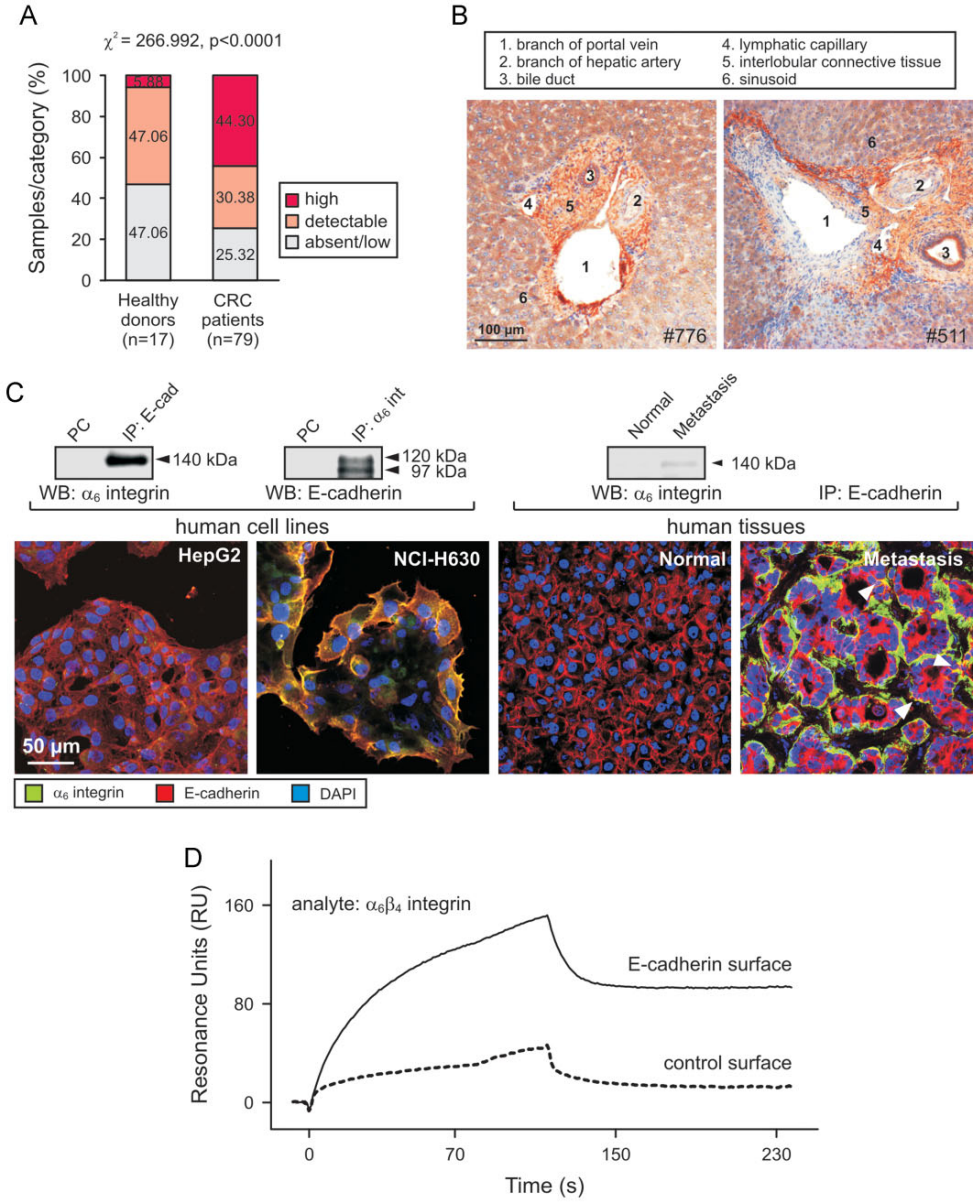
Angiopoietin-like 6 is enriched in hepatic vessels of patients with metastatic CRC, and α_6_ integrin and E-cadherin are part of a molecular complex in liver metastases from CRC **A,B.** The localization of angiopoietin-like 6 was evaluated on 5-µm sections of paraffin-embedded normal livers from 17 healthy donors and grossly normal livers from 79 CRC patients. Two independent pathologists assigned an intensity score for angiopoietin-like 6 staining in blood vessels, and the differences between sample categories were evaluated by a Chi-squared test (**A**). Exemplary pictures of two grossly normal liver samples from patients with metastatic CRC immunostained for angiopoietin-like 6 are shown (**B**).**C.** NCI-H630 and HepG2 were cultured on glass slides for 24 h, followed by immunostaining for E-cadherin and α_6_ integrin. The interaction between these proteins was confirmed by co-IP: 10 mg of total NCI-H630 protein was subjected to IP with anti-E-cadherin or anti-α_6_ integrin antibody, and proteins were separated by 10% SDS–PAGE. Blotted PVDF membranes were exposed to anti-α_6_ integrin or anti-E-cadherin antibody, respectively. Proteins eluted from the preclearing step (PC) were loaded as specificity controls. The localization of α_6_ integrin and E-cadherin was also evaluated on 10-µm sections of OCT-frozen tissue pairs from CRC patients; arrowheads indicate colocalization. A representative staining of normal liver tissue is shown. The interaction between α_6_ integrin and E-cadherin was confirmed by co-IP: 4 mg of total protein from five pooled samples of grossly normal liver and liver metastases was subjected to IP with anti-E-cadherin antibody, and proteins were separated by 10% SDS–PAGE. The blotted PVDF membrane was incubated with anti-α_6_ integrin antibody.**D.** The interaction between α_6_ integrin and E-cadherin was evaluated by SPR analysis. Sensorgrams report the binding of α_6_ integrin (100 nM) to BIAcore CM5 sensor chips coated with either E-cadherin (black line) or VEGFR2 (dotted line, control surface). The response was recorded in RU as a function of time.

We next asked whether angiopoietin-like 6 was differentially expressed in grossly normal livers from metastatic CRC patients (*n* = 79) in comparison to livers from healthy donors (*n* = 17; Supporting Information Fig S1). Although the overall staining for angiopoietin-like 6 in hepatocytes was similar, this ligand significantly accumulated in hepatic blood vessels of cancer patients in comparison to those of healthy individuals (Fig 3A). A closer histological evaluation of normal liver samples from patients with metastatic CRC revealed that angiopoietin-like 6 is present in large blood vessels as well as in capillaries, sinusoids and lymphatics, all potential sites for the molecular recognition of circulating CRC cells through specific ligand/receptor interactions (Fig 3B).

### Alpha 6 integrin and E-cadherin are receptors for an angiopoietin-like 6-mimicking peptide and participate in a supramolecular complex in human liver metastasis secondary to CRC

We produced soluble CGIYRLRSC as a fusion peptide with glutathione S-transferase (CGIYRLRSC-GST) to exploit its interaction with NCI-H630 cell surfaces (Fig 1B) towards the identification of potential receptors. HepG2 cells, which do not bind the CGIYRLRSC-phage (Fig 1B), served as a negative control. We incubated CGIYRLRSC-GST with both cell lysates, followed by separation of the bound proteins by sodium dodecyl sulfate–polyacrylamide gel electrophoresis (SDS–PAGE). Specific bands (*n* = 11) were analysed by mass spectrometry; 37 proteins with an identification score >50 were thereby obtained. Of this set, five cell-surface proteins (*i.e.* putative receptors) and 32 cytoskeletal proteins were found, a result indicating that a protein (or protein complex) involved in cell adhesion and/or motility might be responsible for this interaction. Among the cell adhesion proteins found, α_6_ integrin and E-cadherin exhibited the highest identification score (Supporting Information Table S2).

We first evaluated the localization of α_6_ integrin and E-cadherin in HepG2 and NCI-H630 cells by confocal microscopy. There was colocalization of these proteins in the liver metastasis cell line NCI-H630, in which both α_6_ integrin and E-cadherin were highly represented on the cell membranes. In contrast, barely detectable immunostaining of α_6_ integrin and no colocalization with E-cadherin were observed in the primary tumour cell line HepG2. We further asked whether these proteins could be part of a supramolecular complex in liver metastasis cells, as indicated by mass spectrometry and by their colocalization on the cell surface. Co-immunoprecipitation (IP) assays confirmed that α_6_ integrin and E-cadherin physically interact in NCI-H630 cells (Fig 3C). Confocal imaging analyses performed on liver metastases from CRC patients (*n* = 6) revealed that α_6_ integrin and E-cadherin were expressed by selected groups of cells, with regions of overlap; in contrast, α_6_ integrin was barely detectable and the two proteins did not colocalize in matched grossly normal livers. A co-IP assay performed on proteins from five pooled liver metastases confirmed the presence of an interaction between α_6_ integrin and E-cadherin in these tissues; this interaction could not be detected in samples of grossly normal liver from the same patients (Fig 3C). These results show that α_6_ integrin and E-cadherin are expressed and colocalize in regions of human liver metastases, where they are part of a molecular complex. To elucidate whether α_6_ integrin and E-cadherin interact with each other on the outside of the cells, and to quantify their binding affinity, we performed surface plasmon resonance (SPR) analyses using their recombinant extracellular portions. For this purpose, E-cadherin was immobilized on a BIAcore CM5 sensor chip; a reference chip was prepared by immobilization of the vascular endothelial growth factor receptor 2 (VEGFR2). These analyses confirmed that the interaction of α_6_ integrin with E-cadherin is specific (Fig 3D). Although it was not possible to perform dose–response measurements on a single sensor chip (see Materials and Methods Section for details), repeated injections of α_6_ integrin on newly prepared E-cadherin-coated surfaces provided values of dissociation constant (*K*_d_) ranging from 5.3 to 16.8 nM. These results demonstrate that the extracellular domains of α_6_ integrin and E-cadherin bind directly to each other and with a relatively high affinity.

### A supramolecular complex of α_6_ integrin and E-cadherin is the receptor for angiopoietin-like 6

**Figure 4:**
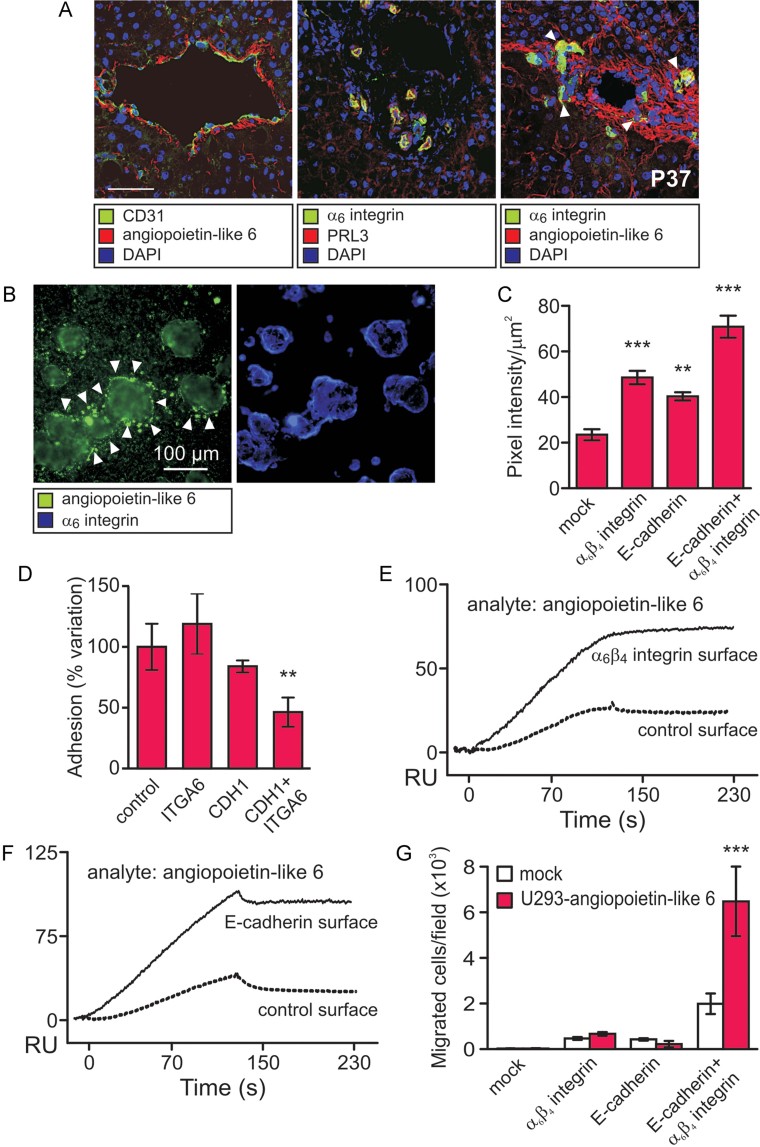
Angiopoietin-like 6 interacts with α_6_ integrin and E-cadherin **A.** The interaction of angiopoietin-like 6 with micrometastases was evaluated on 10-µm frozen sections of grossly normal liver tissues from three CRC patients. Endothelial cells were identified by immunostaining for CD31, and metastatic cells by immunostaining for PRL3 and α_6_ integrin. Immunostaining for angiopoietin-like 6 and α_6_ integrin was performed to investigate the colocalization of hepatic angiopoietin-like 6 and metastatic cells. Representative images of tissues from two patients are shown; arrowheads indicate colocalization.**B.** U293 cells transduced with α_6_ integrin and E-cadherin were mixed with equal amounts of cells expressing angiopoietin-like 6 and cultured on glass slides for 48 h, before staining for angiopoietin-like 6 and α_6_ integrin.**C,D.** U293 cells transfected with an empty vector (mock) or with the cDNAs coding for the receptor proteins were grown on glass slides for 24 h before incubation with conditioned media from angiopoietin-like 6-secreting cells. Bound ligand was revealed by fluorescence immunostaining, quantified as mean pixel intensity and expressed as percent variation compared to incubations with supernatants of mock-transfected cells (**C**). NCI-H630 cells silenced for *ITGA6*, *CDH1* or both mRNAs were incubated onto angiopoietin-like 6-coated microwells. Numbers of adhered cells are expressed as percent variation compared to wild-type cells (**D**).**E,F.** The interaction between angiopoietin-like 6 and its receptors was evaluated by SPR analysis. Sensorgrams report the binding of angiopoietin-like 6 (200 nM) to BIAcore NTA sensor chips coated with α_6_β_4_ integrin (**E**), E-cadherin (**F**) (black lines) or to the reference, nickel-saturated surfaces (dotted lines). The response was recorded in RU as a function of time.**G.** U293 cells transduced with α_6_ integrin, E-cadherin or both were co-cultured with cells expressing angiopoietin-like 6. After 48 h, cells on the lower side of the transwell membranes were fixed, stained and counted under a light microscope at 5× magnification. In **C**, **D** and **G**, results are shown as mean ± standard deviation for each experimental point in two independent experiments. Asterisks indicate the following *p*-value ranges: **p < 0.01, ***p < 0.001.

In a small subset of samples of grossly normal liver, we observed a few cellular aggregates that were positive for the metastatic marker phosphatase of regenerating liver 3 (PRL3; Saha et al, 2001) and for α_6_ integrin. Such micrometastatic foci were associated with blood vessels, on which an extensive colocalization of hepatic angiopoietin-like 6 and metastatic α_6_ integrin was evident (Fig 4A).

To design a molecularly defined cell model capable of reproducing these interactions, we prepared U293 cell clones stably expressing (i) α_6_ integrin, E-cadherin or both (receptors, metastasis side), or (ii) angiopoietin-like 6 (ligand, host tissue side) (Supporting Information Fig 2A). In a first set of experiments, we noticed that cells transduced with both receptors, when mixed with cells secreting the ligand, segregate into metastasis-like aggregates surrounded by soluble angiopoietin-like 6 and in tight contact with angiopoietin-like 6-transduced cells (Fig 4B).

For an accurate evaluation of this ligand/receptor interaction, we incubated α_6_ integrin-, E-cadherin- or α_6_ integrin/E-cadherin-transduced cells with conditioned media from angiopoietin-like 6-secreting cells, in which the protein was detected by ELISA at a concentration ranging from 60 to 80 µg/ml. Mock-transfected cells served as a reference for background binding, and corresponding supernatants were used as negative controls. Confocal microscopy quantification of cell surface-associated ligand revealed that angiopoietin-like 6 was capable of interacting with cells expressing either α_6_ integrin or E-cadherin; this interaction was strong when both receptors were present (Fig 4C). To confirm these data, we performed siRNA-mediated down-regulation of α_6_ integrin (*ITGA6*), E-cadherin (*CDH1*) or both mRNAs (Supporting Information Fig S3A) in NCI-H630 cells, and we evaluated the capacity of these cells to interact with angiopoietin-like 6. In this assay, NCI-H630 cells in which both *ITGA6* and *CDH1* mRNAs were down-modulated exhibited impaired adherence to microwells coated with recombinant angiopoietin-like 6 (Fig 4D). These results suggest that hepatic angiopoietin-like 6 represents a potential, thus far unrecognized ligand for metastatic cells that express both α_6_ integrin and E-cadherin. To investigate these interactions in detail, we again performed SPR analyses using all recombinant proteins. Taking advantage of their his-tag, the extracellular portions of E-cadherin and α_6_ integrin were properly oriented and immobilized on nickel-activated BIAcore NTA sensor chips. A chip saturated with nickel was used as a reference. These analyses demonstrated that angiopoietin-like 6 binds specifically to both receptors (Fig 4E–F). Dose–response measurements (data not shown) allowed calculating *K*_d_ values for the interaction with α_6_ integrin and E-cadherin equal to 2.03 and 29.0 nM, respectively.

Angiopoietin-like 6 is a secreted factor with chemotactic activity on endothelial cells (Oike et al, 2004). We asked whether this ligand could also affect the motility of cells expressing α_6_ integrin and E-cadherin. U293 cells transduced with α_6_ integrin, E-cadherin or both were co-cultured with angiopoietin-like 6-secreting cells in a transwell system, and their migration towards the ligand gradient was evaluated. Co-cultures with mock-transfected U293 cells were used as a reference for basal cell motility. Although the presence of either α_6_ integrin or E-cadherin slightly increased the migration of U293 cells under basal conditions, this feature was not influenced by angiopoietin-like 6. In contrast, U293 cells expressing both α_6_ integrin and E-cadherin showed a basal migratory phenotype that was stimulated significantly by angiopoietin-like 6 (Fig 4G). These data demonstrate that the presence of both α_6_ integrin and E-cadherin is necessary to respond to a gradient of angiopoietin-like 6, further confirming a functional role for this ligand/receptor system.

### CRC cell adhesion to the hepatic tissue *in vitro* and liver colonization *in vivo* are mediated by α_6_ integrin and E-cadherin and are inhibited by an angiopoietin-like 6-mimicking peptide

**Figure 5:**
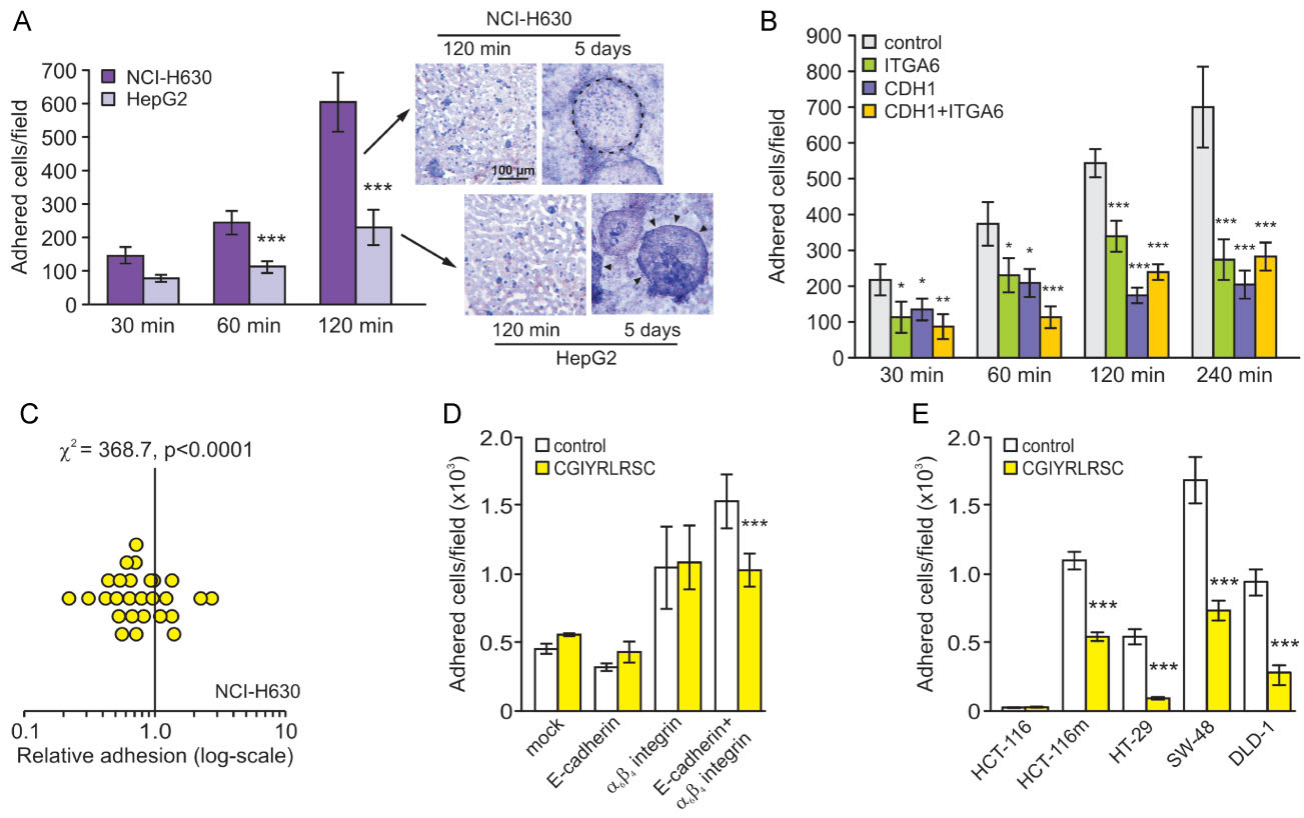
Alpha 6 integrin/E-cadherin-mediated liver adhesion is counteracted by an angiopoietin-like 6-mimicking peptide **A,B.** HepG2 and NCI-H630 were incubated on 10-µm frozen sections of grossly normal liver from CRC patients, and adhered cells were fixed, haematoxilin-stained and counted under a light microscope at 20× magnification. Photomicrographs representative of cell numbers (120 min) and morphology (5 days) are shown. The dotted line indicates a micrometastasis-like structure integrated into the liver tissue; arrowheads point to cell aggregates (**A**). NCI-H630 cells silenced for *ITGA6*, *CDH1* or both mRNAs were challenged in the same assay (**B**). Results are shown as mean ± standard deviation for each experimental point in three independent experiments.**C–E.** NCI-H630 cells (**C**), U293 cells transduced with α_6_ integrin, E-cadherin or both (**D**) and CRC cell lines (**E**) were incubated with 10-µm frozen sections of grossly normal liver in the presence of either control or CGIYRLRSC peptide. After 30 min, adhered cells were fixed, stained and counted under a light microscope at 5× magnification. For NCI-H630 cells, adhesion was evaluated on sections from 27 patients and results are shown as the ratio of attached cells comparing CGIYRLRSC and control peptide incubations. In **A**, **B**, **D** and **E**, differences in the experimental points were evaluated for their statistical significance by ANOVA followed by Bonferroni's post-test. In **C**, a Chi-squared test was used to evaluate whether values were significantly different from 1.

To evaluate a potential role for the described system in the process of liver metastasis, we first compared the capacity of HepG2 and NCI-H630 cells to adhere to grossly normal livers from CRC patients (a characterization of these cell lines in terms of CGIYRLRSC-phage binding and of α_6_ integrin/E-cadherin expression and coexpression is reported in Fig 1B and Fig 3C, respectively). Cells were incubated on human liver sections for increasing periods of time, ranging from 30 min to 5 days. At all time points, HepG2 cells adhered weakly to the liver and grew in separate aggregates. In contrast, the adhesion of NCI-H630 cells was significantly higher; moreover, these cells could grow and integrate into the hepatic tissue (Fig 5A), with a morphology reminiscent of the metastasis model described in Fig 4A. This effect was modulated by α_6_ integrin and E-cadherin, as confirmed by the fact that cells in which *ITGA6*, *CDH1* or both mRNAs were down-modulated exhibited impaired adherence to normal liver, with the double-silenced cells showing the slowest adhesion (Fig 5B).

Because the insert in the CGIYRLRSC sequence mimics a liver-enriched ligand for α_6_ integrin and E-cadherin, we investigated whether the corresponding peptide could interfere with this interaction, and thereby inhibit the adhesion of metastatic cells to the hepatic tissue. When NCI-H630 cells were incubated on grossly normal liver sections from CRC patients (*n* = 27), there was a significant decrease in cell adhesion upon treatment with a soluble CGIYRLRSC peptide (Fig 5C). We also investigated the adherence to grossly normal liver of U293 cells stably transduced with cDNAs encoding for α_6_ integrin, E-cadherin or both (Fig 5D). U293 cells in which α_6_ integrin was expressed, either alone or in combination with E-cadherin, showed an increased affinity for the hepatic tissue; liver adhesion was the most pronounced in cells expressing both components of the receptor complex. Notably, CGIYRLRSC inhibited adhesion of U293 cells only when they expressed both α_6_ integrin and E-cadherin. We finally evaluated the adhesive properties of different cell lines of primary CRC with proven *in vivo* metastatic behaviour, *e.g.* HCT-116m, HT-29 (Price et al, 1989), SW-48 and DLD-1 (Tibbetts et al, 1993; Fig 5E, Supporting Information Fig 2B). These cells exhibited remarkable adhesion to grossly normal liver, which was significantly inhibited by CGIYRLRSC. The non-metastatic version of HCT-116 cells, which express very low levels of the receptor proteins, adhered poorly to the hepatic tissues and was not affected by CGIYRLRSC. These results suggest that CGIYRLRSC-mimicking proteins, such as angiopoietin-like 6, can act as microenvironment addresses for metastatic cells that express α_6_ integrin and E-cadherin.

**Figure 6:**
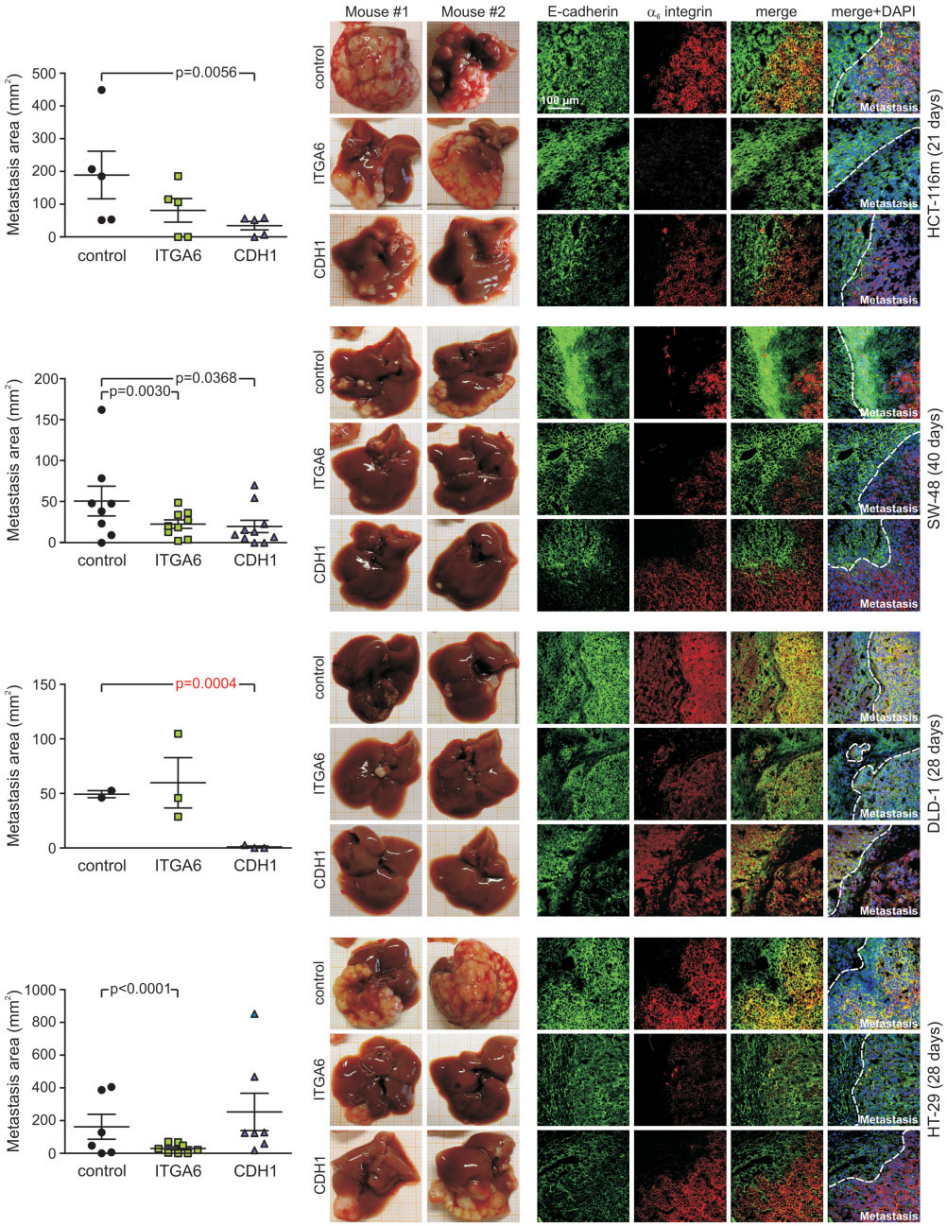
Impaired expression of α_6_ integrin or E-cadherin results in impaired liver colonization *in vivo* HCT-116m, SW-48, DLD-1 and HT-29 cell lines with down-modulated expression of α_6_ integrin or E-cadherin were injected intrahepatically into CD-1 nude mice (6–10 mice/group, 5 × 10^6^ cells/mouse). At the indicated time points, mice were euthanized, and their livers were explanted and photographed for the quantification of external tumour areas. Representative pictures of whole livers from 2 mice/group are shown for macroscopic evaluation of tumour morphology; the indicated *p*-values are referred to statistical analysis performed with Fisher's exact test (black) or *t*-test (red). Sample tissues were OCT-frozen, cut in 10-µm sections, and immunostained with anti-α_6_ integrin and anti-E-cadherin antibodies, followed by imaging with a confocal microscope. Acquisition parameters were held constant to allow comparison of the absolute amounts and localizations of α_6_ integrin and E-cadherin among the different samples. Exemplary pictures of samples from 1 mouse/group are shown.

The data obtained *in vitro* prompted us to ask whether similar mechanisms would be involved in hepatic colonization *in vivo*. To study the metastasis/host tissue interaction, we established an animal model of hepatic colonization by direct injection of human CRC cell lines into the livers of CD-1 nude mice. For a molecular analysis of the receptor side, we achieved stable shRNA-mediated silencing of *ITGA6* or *CDH1* mRNA in different cell lines of primary CRC, *e.g.* HCT-116m, HT-29, SW-48 and DLD-1 (Supporting Information Fig S3B). All the cells in which either component of the receptor complex was silenced exhibited an impaired capability to form tumours after injection into the liver; this effect reached statistical significance in SW-48 and HT-29 cells in which α_6_ integrin was silenced, and in HCT-116m, SW-48 and DLD-1 cells in which E-cadherin was silenced. By confocal microscopy we confirmed that the decreases in specific proteins were retained *in vivo*, with an almost complete disappearance of coexpressed α_6_ integrin and E-cadherin from the tumour tissues (Fig 6). These data show that even an incomplete depletion of only a receptor protein is sufficient to alter liver colonization by CRC cells.

**Figure 7:**
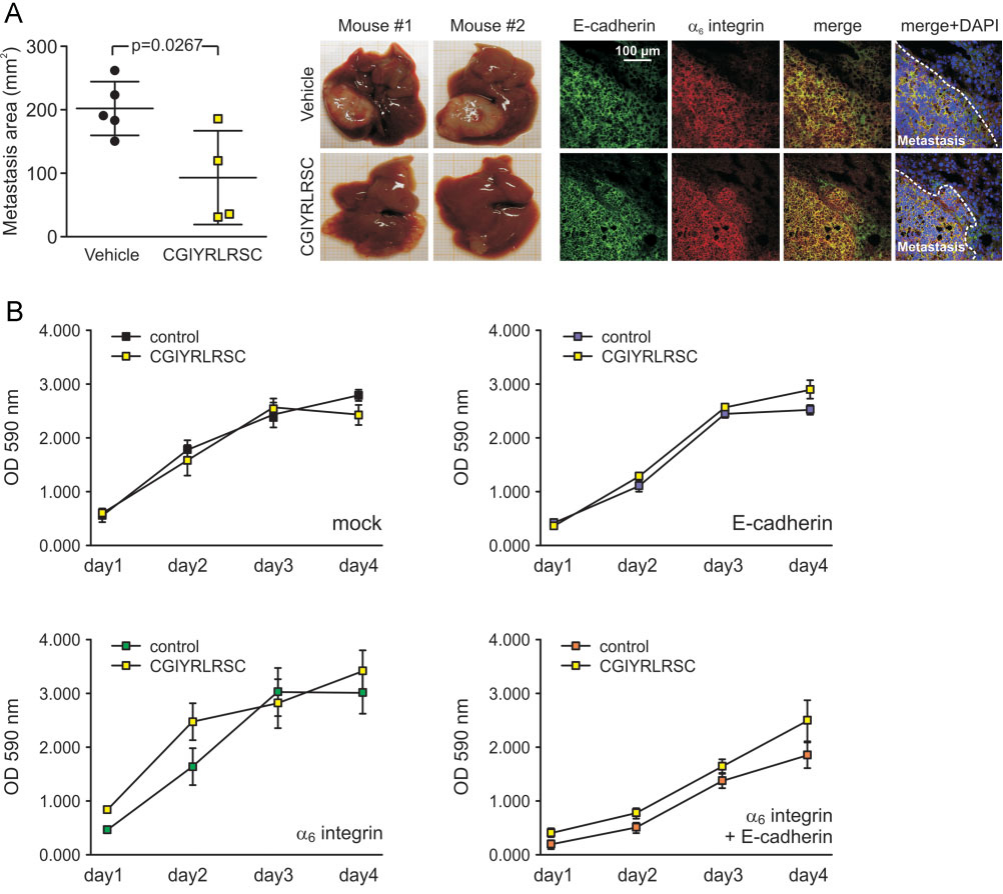
An angiopoietin-like 6-mimicking peptide interferes with liver colonization but not with the proliferation of human metastatic CRC cells CGIYRLRSC interferes with hepatic colonization *in vivo*. LS-174T cells were injected intrahepatically into CD-1 nude mice (6 mice/group, 5 × 10^6^ cells/mouse), either in medium alone (vehicle) or in the presence of CGIYRLRSC. Fourteen days after surgery, animals were euthanized and tissues were processed as described in Fig 6. The indicated *p*-value is referred to statistical analysis performed with Fisher's exact test.U293 cells expressing E-cadherin, α_6_ integrin or both were grown in complete culture medium, in the presence of either control or CGIYRLRSC peptide. At 24 h time points, cells were fixed and stained with crystal violet; their numbers were estimated by spectrophotometric evaluation. Results are shown as mean ± standard deviation for each experimental point in three independent experiments.

We next evaluated whether interference with the described ligand/receptor pair would lead to impaired hepatic colonization. For this set of experiments we used LS-174T, a cell line derived from a primary CRC that exhibits high expression of the complex proteins (Supporting Information Fig 2B) and an aggressive behaviour *in vivo* (Tibbetts et al, 1993). We injected animals with LS-174T cells either in medium alone or in the presence of CGIYRLRSC. After 14 days we explanted the livers for tumour quantification. We observed a significant reduction of liver tumours in mice injected with LS-174T cells in the presence of the angiopoietin-like 6-mimicking peptide, although the overall morphology and the levels of α_6_ integrin and E-cadherin, as well as their colocalization, were unchanged (Fig 7A). These results indicate that CGIYRLRSC might interfere with early steps of tumour/host tissue recognition, without influencing successive tumour growth. Consistently, CGIYRLRSC has no effects on the proliferation of U293 cells transduced with α_6_ integrin, E-cadherin or a combination of both receptors (Fig 7B).

### An angiopoietin-like 6-mimicking peptide has antimetastatic effects in preclinical models of human CRC

**Figure 8:**
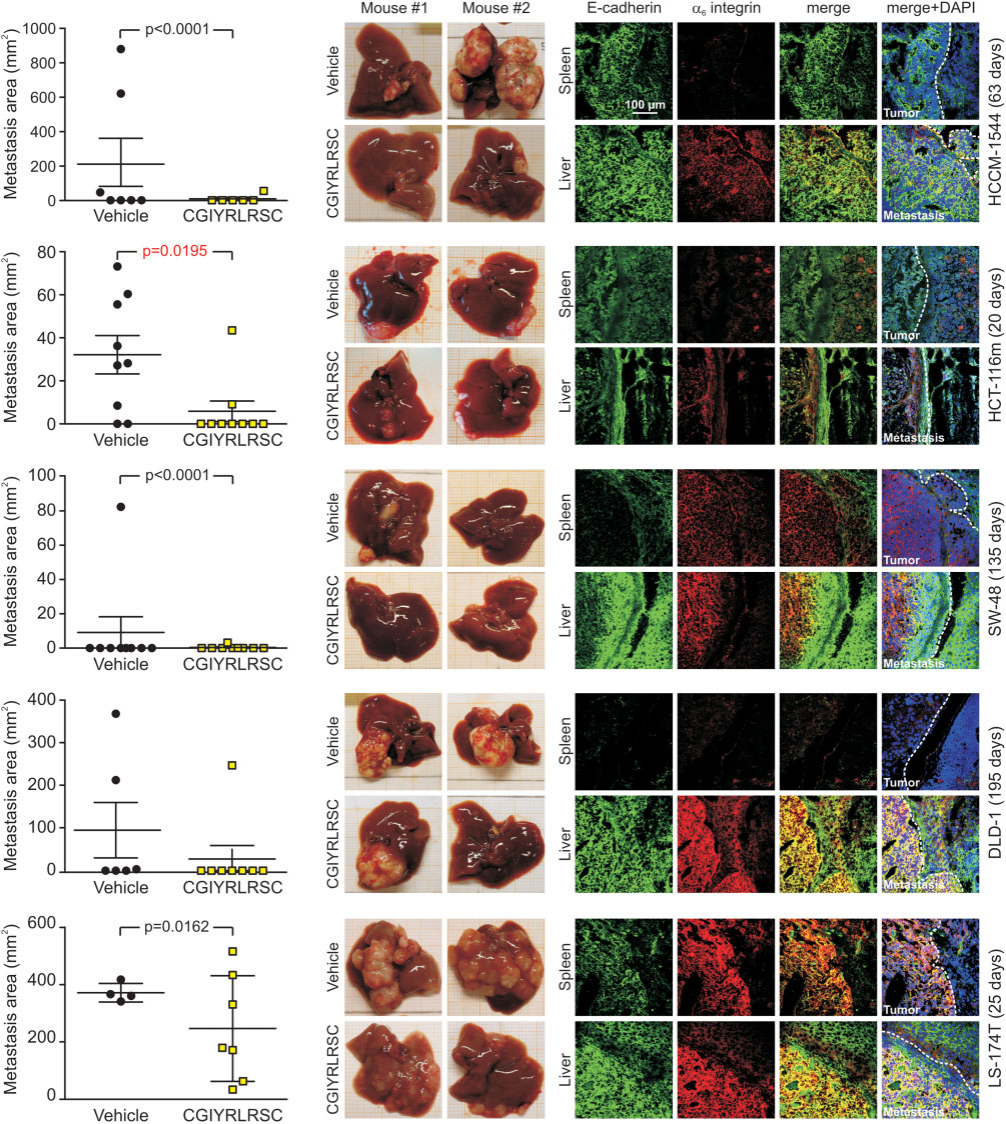
An angiopoietin-like 6-mimicking peptide inhibits homing of CRC cells to the liver To obtain an *in vivo* pseudo-orthotopic model of metastatic CRC, we implanted the human tumour HCCM-1544, and the cell lines HCT-116m, SW-48, DLD-1 and LS-174T, intrasplenically into CD-1 nude mice (6–10 mice/group, 2 × 10^6^/mouse). Cells were injected in medium alone (vehicle) or in the presence of CGYIRLRSC. At the indicated time points, mice were euthanized, and tissues were processed as described in Fig 6.

Having demonstrated that *in vitro* angiopoietin-like 6 is a potent chemoattractant for cells expressing α_6_ integrin and E-cadherin (Fig 4F), we asked whether the angiopoietin-like 6-mimicking peptide CGIYRLRSC could interfere with the homing of CRC cells to the liver *in vivo*. To obtain a pseudo-orthotopic preclinical model of metastatic CRC, we implanted the human tumour HCCM-1544 (Giavazzi et al, 1986), as well as a panel of human cell lines (HCT-116m, SW-48, DLD-1 and LS-174T) into the spleens of CD-1 nude mice. Cells were injected either in medium alone or in the presence of CGIYRLRSC. Mice were euthanized at different time points after cell injection, from 20 (HCT-116m) to 195 days (DLD-1), reflecting the different aggressiveness of the cell lines. At the time of sacrifice, in all the tumour models a primary splenic mass and a variable number of liver metastases were present; the frequency of hepatic involvement in the vehicle group varied from 11% (DLD-1) to 100% (LS-174T). Treatment with CGIYRLRSC resulted in diminished homing of CRC cells to the liver, and was significant in all the models investigated, with the exclusion of the poorly metastatic DLD-1 cell line (Fig 8). These data confirm that CGIYRLRSC inhibits liver homing of CRC cells independently of the characteristic features (timing, size and number of metastases) of each model. In all the experimental settings, we observed a substantial increase in both α_6_ integrin and E-cadherin, with diffuse regions of colocalization, in liver metastases as compared to primary spleen tumours (Fig 8). This result suggests that the expression of such proteins is augmented and/or that highly expressing cells are selected during metastatic progression.

### The levels of α_6_ integrin/E-cadherin coexpression correlate with the progression of human metastatic CRCs

**Figure 9:**
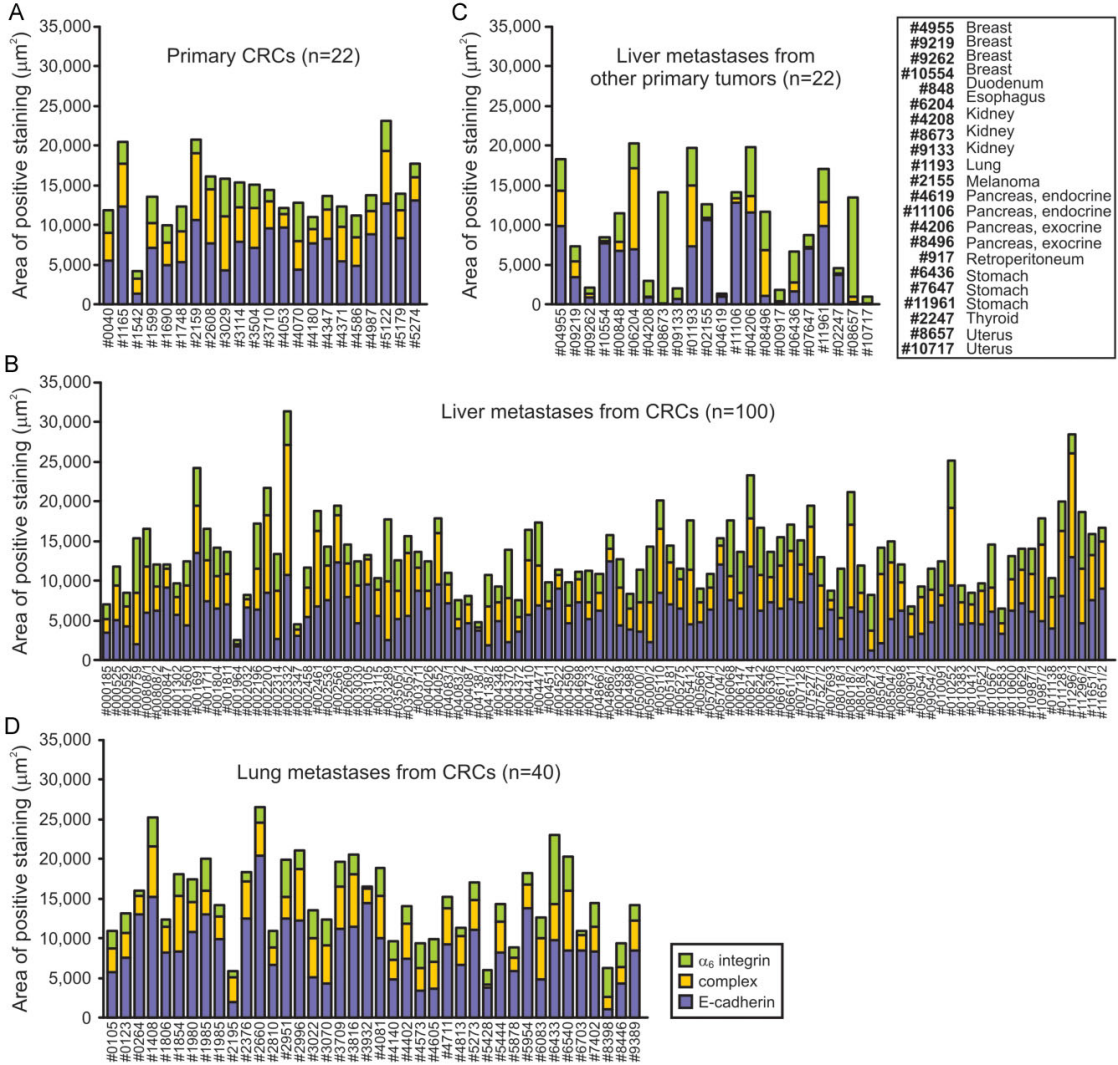
α_6_ Integrin, E-cadherin and their molecular complex are associated to advanced CRCs **A–D.** The presence of α_6_ integrin and E-cadherin was evaluated by immunostaining of 5-µm sections of a panel of paraffin-embedded cancer tissues (**A**, primary CRCs; **B**, Liver metastases from CRCs; **C**, liver metastases from other primary tumours, the origin of which is indicated in the table; and **D**, lung metastases from CRCs). The quantification of specific fluorescent signals is described in the Material and Methods Section.

**Figure 10:**
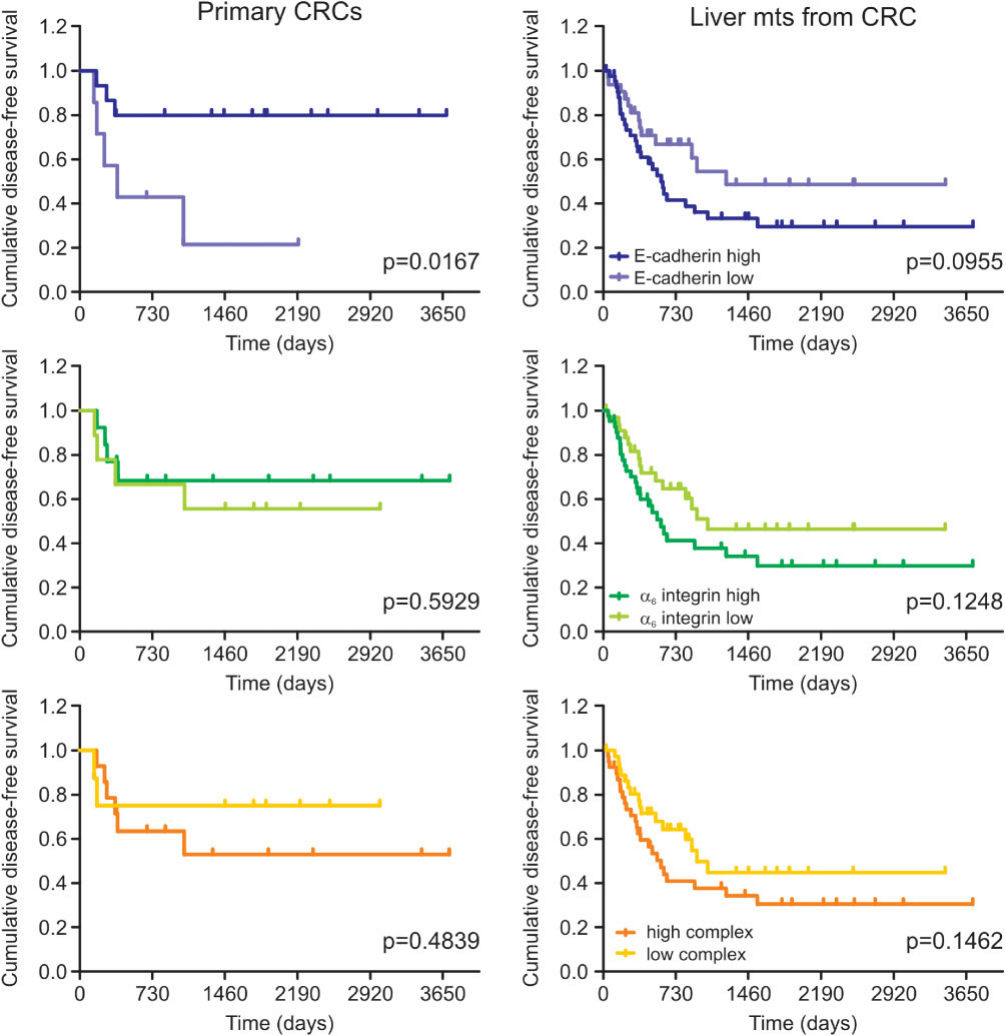
The coexpression of α_6_ integrin and E-cadherin is a poor prognostic factor for patients with metastatic CRC The amounts of α_6_ integrin, E-cadherin, and their molecular complex in primary CRCs (grade IV) and liver metastases from CRCs were correlated with disease-free survival. Survival curves were drawn as Kaplan–Maier Cumulative Proportion Surviving graphs and corresponding *p*-values were calculated by the use of the log-rank (Mantel-Cox) test with Prism 5 GraphPad software.

We finally evaluated the role of the described ligand/receptor system from a clinical perspective. By quantitative confocal imaging, we evaluated the amounts of α_6_ integrin, E-cadherin and their colocalization in the following tumour settings: primary CRCs (Duke's stage IV; *n* = 22; Fig 9A), liver metastases from CRC (*n* = 100; Fig 9B), liver metastases from other cancers (*n* = 22; Fig 9C), and lung metastases from CRCs (*n* = 40; Fig 9D). This analysis revealed that the presence of the α_6_ integrin/E-cadherin complex is constant in advanced CRCs, from primary adenocarcinomas to liver and lung metastases. Conversely, liver metastases from different primary tumours exhibited variable expression of α_6_ integrin and E-cadherin, which resulted in their colocalization in <50% of the samples examined. A correlation analysis with the clinical outcome of CRC patients revealed that high levels of α_6_ integrin, E-cadherin, and their coexpression in liver metastases were all associated with shorter disease-free survival after surgical intervention (Fig 10). In primary adenocarcinomas, despite an inverse correlation with the levels of E-cadherin and lack of correlation with respect to α_6_ integrin, high amounts of colocalized α_6_ integrin and E-cadherin were nevertheless associated with shorter disease-free survival (Fig 10). These data indicate that the amounts of coexpressed α_6_ integrin/E-cadherin might be exploited as a new prognostic factor to identify a subset of metastatic CRCs characterized by fast disease progression.

## DISCUSSION

Understanding the early steps of metastasis is crucial from a therapeutic perspective, because at this stage specific molecular mechanisms likely define the metastatic destination of cancer cells, and therefore targeted inhibitors could make the difference in a patient's prognosis. Although studies have been ongoing since Paget first proposed the “seed and soil” hypothesis (Paget, 1889), many questions remain. For breast cancer, a match between tumour-expressed receptors and host tissue-expressed chemokines appears to be the driving force for the onset of metastasis. In this pathological context, specific signalling pathways have recently emerged that are selectively involved in lung (Minn et al, 2005) or brain (Bos et al, 2009) metastasis. For CRC, with the exception of the specific liver colonization of metastatic cells (Kuo et al, 1995) and site-dependent variations in therapeutic responses (Dong et al, 1994), little is known about the mechanisms that drive hepatic homing and colonization. Indeed, current therapies for advanced CRC target molecules that are related to cancer progression [*e.g.* VEGF (Grothey & Galanis, 2009) and EGFR (Okamoto, 2009)], rather than counteract the initiation of metastasis.

The difficulty in addressing the metastatic process resides in the high variability of cancer cells, a consequence of several events that occur in the life history of a tumour. Different hypotheses have been debated for decades that focus on at least two mechanisms providing valuable models for metastasis progression. The first is the selection and expansion of metastatic clones preexisting in the primary tumour (Talmadge, 2007), which was demonstrated in mouse models (Fidler & Kripke, 1977) and subsequently confirmed in patients (Klein et al, 2002). The second is adaptation of cancer cells to signals from recruited (normal) stromal cells (Condeelis & Pollard, 2006; Scheel et al, 2007). Adaptation mechanisms have been observed both in primary tumours, in which malignant cells exploit contextual signals to undergo the epithelial–mesenchymal transition (Thiery & Sleeman, 2006), as well as in distant metastases, where the opposite transition may occur (Chaffer et al, 2006). In some cases, adaptation is made easier: some microenvironments show an intrinsic or preinduced permissiveness for colonization by circulating cancer cells (Coghlin & Murray, 2010; Peinado et al, 2011; Talmadge, 2010). It is now clear that composite events are exploited by cancer cells for their malignant progression, and that Darwinian selection of genetic/epigenetic variants is complemented by opportunistic adaptation to the host physiology (Hanahan & Weinberg, 2011). In this view, a system of tumour/host attraction and colonization will be greatly favoured, provided that it meets at least one of the following requirements.

The first one is profiting by preexisting components. Here we describe a molecular mechanism for liver metastasis that is based on a liver-enriched, naturally occurring ligand, angiopoietin-like 6 and on receptors already present in primary CRCs, α_6_ integrin and E-cadherin. As proof of this aspect of the adaptation hypothesis, the accumulation of angiopoietin-like 6 in liver vessels is significantly increased in patients with metastatic CRC in comparison to healthy individuals. Vessel-enriched angiopoietin-like 6 might be secreted by (primary or metastatic) tumour cells; alternatively, it might be induced in normal liver cells by tumour-derived factors. The consequent increase in docking sites for circulating CRC cells can be further accompanied by the onset of autocrine circuits in malignant cells themselves. Furthermore, we demonstrate that (i) subsets of cells characterized by high levels of coexpressed α_6_ integrin and E-cadherin are enriched during the progression from primary tumour to hepatic metastasis in animal models, and (ii) patients whose primary adenocarcinomas express high amounts of this receptor exhibit a shorter disease-free survival. Although more extensive investigation is needed to elucidate the physiopathological mechanisms, these data are reminiscent of the pro-metastatic systemic perturbation described by Weinberg and coworkers (McAllister & Weinberg, 2010).

The second requirement relates to the adaptability of protein–protein interactions. In addition to mutation and/or amplification of oncogenic molecules, a driving mechanism in cancer progression appears to be the emergence of unusual protein partners whose association results in altered biological activities. For example, E-cadherin has been shown to interact directly with α_E_β_7_ integrin to mediate heterotypic adhesion between T-lymphocytes and epithelial cells under physiological (Cepek et al, 1994) and pathological (Le Floc'h et al, 2007) conditions. In a CRC cell line, E-cadherin interacts with insulin-like growth factor-I receptor and α_v_ integrin; upon ligand stimulation, this complex is destroyed and the integrin is relocalized from intercellular contacts to focal adhesions, a condition leading to increased cell motility (Canonici et al, 2008). We show for the first time that a heretofore unrecognized complex of E-cadherin and α_6_ integrin is present on the surface of CRC cells, with a functional role in liver homing and colonization. This mechanism could be exploited by other tumours that metastasize to the liver, as suggested by the presence of this complex in ∼50% of the non-CRC liver metastases that we have investigated.

A third requirement exploits modular interactomes. Degenerate patterns of protein–protein recognition can build up dynamic networks. As an example, RGD is a recognition motif shared by several integrin substrates, whose flanking regions confer affinity for receptor binding (Humphries et al, 2006). Such a system opens a plethora of possibilities for a parasite cell to find potential molecular partners in a host microenvironment; it is therefore not surprising that our biopanning experiments resulted in the enrichment of conserved peptide motifs as ligands for metastatic CRC cells. Indeed, besides angiopoietin-like 6, a number of extracellular proteins share similarity with the metastasis-specific peptides, among which a proteoglycan (perlecan) and a component of the basal lamina (laminin α_2_), both involved in angiogenesis and cancer progression (Theocharis et al, 2010; Vitolo et al, 2006). A further ligand emerging from this search was osteopontin, a secreted phosphoprotein with a recognized role in metastatic CRC. Interestingly, in the primary sequence of osteopontin, the LRS motif is only five amino acids beyond the RGD domain. The region starting with LRS is proteolytically cleaved by metalloproteinase-9, to release a fragment in which a cryptic adhesive sequence is revealed that exhibits altered affinity for integrins and acquires pro-metastatic activity (Green et al, 2001; Smith et al, 1996; Takafuji et al, 2007).

Taken together, these observations strengthen our suggestion that the angiopoietin-like 6/α_6_ integrin/E-cadherin system is part of an interconnected network, in which several proteins interact with different molecular partners based on their availability, combination and affinity. It is possible that a targeted approach mediated by CGIYRLRSC or other metastasis-binding peptides might interfere with a number of protein–protein interactions based on this pattern recognition, thereby resulting in an amplification of the therapeutic outcome. The fact that extracellular proteins share common features has complicated the elucidation of their interactions with the single-molecule approaches used in the recent past. Conversely, we demonstrate that combinatorial profiling can lead to the identification of functional addresses as putative molecular targets within the microenvironment of human hepatic metastasis (Marchiò et al, 2009). In this case, the presence of overlapping protein motifs will facilitate targeting the metastatic site even without specific knowledge of each participant.

## MATERIALS AND METHODS

### Antibodies, proteins, peptides

Goat polyclonal anti-α_6_ integrin N-19 (for immunoblot) and anti-vinculin N-19, rabbit polyclonal anti-β_4_ integrin H-101 (for ELISA) (sc-9090) and horseradish peroxidase (HRP)-conjugated anti-goat IgG were obtained from Santa Cruz Biotechnology (Santa Cruz, CA). Mouse monoclonal anti-α_6_ integrin clone BQ16 (used for IP) was obtained from Calbiochem (San Diego, CA). Rat monoclonal anti-α_6_ integrin clone GoH3 (for immunostaining) was obtained from AbD Serotec (Raleigh, NC). Mouse monoclonal anti-β_4_ integrin clone 7 (for immunoblot) and anti-E-cadherin clone 36 were obtained from BD Transduction Laboratories (Franklin Lakes, NJ). Rabbit polyclonal anti-fd bacteriophage was obtained from Sigma (St. Louis, MO). Alexa Fluor®488 anti-rat and 555 anti-mouse IgG were obtained from Invitrogen (Carlsbad, CA). HRP-conjugated anti-mouse IgG was obtained from Jackson ImmunoResearch (West Grove, PA). Mouse monoclonal anti-CD31 clone JC70A and HRP-conjugated anti-rabbit EnVision were obtained from DAKO (Glostrup, Denmark). Rabbit polyclonal (for immunostaining) and mouse monoclonal clone Kairos-60 (for immunoblot) anti-angiopoietin-like 6, and angiopoietin-like 6 were obtained from Alexis Biochemicals (Enzo Life Sciences, Farmingdale, NY). Rabbit polyclonal anti-PRL3 was a gift of Dr. Alberto Bardelli (IRCC; Bardelli et al, 2003). CGIYRLRSC and control (CARAC) peptides were obtained from New England Peptides (Gardner, MA). Recombinant Fc-his-tag-E-cadherin, his-tag-α_6_β_4_ integrin, and Fc-his-tag-VEGFR2 (all extracellular portions) were obtained from R&D Systems (Space Import-Export, Milan, Italy).

### Cell lines and human samples

SW480, SW620, NCI-H630, HepG2, AGS, NCI-N87, Capan-2), Capan-1, BT-474, MCF-7, A549, NCI-H1688, HCT-116, HT-29, DLD-1, SW-48, LS-174T and U293 cells were obtained from LGC-Promochem (Sesto San Giovanni, Italy), and were cultured according to the provider's instructions. The HCCM-1544 human metastatic CRC has been previously described (Giavazzi et al, 1986). A variant of HCT-116, selected *in vivo* for its capacity to metastasize to the liver in pseudo-orthotopic models (HCT-116m), was provided by Dr. Alberto Bardelli. Fresh (grossly normal liver from CRC patients, primary CRC, liver metastasis from CRC), and paraffin-embedded human specimens (grossly normal liver from CRC patients, primary CRC, liver metastasis of various origins) were collected by the Units of Surgical Oncology and Pathology at the IRCC and Mauriziano Hospital (Torino, Italy). Paraffin-embedded human specimens of normal liver from healthy donors, of lung metastasis from CRC, and of different healthy tissues were collected by the Unit of Pathology at Molinette Hospital (Turin, Italy). Snap-frozen samples of lung metastasis from CRC and of liver metastasis from renal cancer were obtained from San Luigi Gonzaga Hospital (Orbassano, Italy); snap-frozen samples of ovarian cancer and sigma metastasis were obtained from Mario Negri Institute (Milan, Italy). Collection and manipulation of human samples were approved by the Institutes' Ethical Committees; informed consent was obtained in accordance with the Declaration of Helsinki.

### Phage display

Tissue samples were digested with 0.025% collagenase A (Roche Diagnostics, Monza, Italy) in Iscove's Modified Dulbecco's Minimum Essential Medium (IMDM) for 2 h at 37°C. The resulting suspension was passed through a 40 µm nylon cell strainer (BD Labware, Franklin Lakes, NJ), and cells were resuspended and maintained in binding medium (IMDM supplemented with 2% foetal calf serum, FCS) at 4°C for the duration of the experiments. 10^10^ transducing units (TU) of a CX_7_C, CX_9_C or CX_3_CX_3_CX_3_C phage library was added to 5 × 10^5^ liver metastasis cells and incubated overnight (first round). For successive rounds, phage was preadsorbed on grossly normal liver cells for 1 h at 4°C and then incubated with liver metastasis cells for 2 h at 4°C. Cells were washed five times in binding medium, and bound phage was recovered and amplified by infection of K91Kan *Escherchia coli* bacteria in log-phase. Purification of phage particles and DNA sequencing of phage-displayed inserts were performed as described (Scott & Smith, 1990; Smith & Scott, 1993). Binding on whole cells was performed with 10^9^ TU of each phage on 5 × 10^5^ suspended cells in binding medium as described (Marchiò et al, 2004). For overlay experiments, 5 × 10^9^ TU/ml of each phage was incubated with 10-µm sections of OCT-frozen tissues and detected as described (Arap et al, 2002), with the EnVision system (DAKO) and 3-amino-9-ethylcarbazole (AEC) as substrate. Images were acquired with an EC3 Leica camera (Leica Microsystems, Milan, Italy).

### Identification of peptide-targeted proteins

The following oligonucleotides were annealed and inserted into pGEX-4T.1 between *Bam*HI and *Not*I sites to create the pGEX-4T.1-CGIYRLRSC plasmid:

5′-GATCCGGAGCCTGTGGAATATATAGATTAAGAAGTTGTGCGGGCGC-3′ and
5′-GGCCGCGCCCGCACAACTTCTTAATCTATATATTCCACAGGCTCCG-3′.

The corresponding fusion peptide was purified from BL-21 *E. coli* cell lysates by affinity chromatography on glutathione-sepharose beads (GE Healthcare, Chalfont St. Giles, UK), according to the manufacturer's protocol. HepG2 and NCI-H630 cells were lysed in 50 mM Tris–HCl (pH 7.4), 150 mM NaCl, 0.1% NP-40, 10% glycerol and a protease inhibitor cocktail (Sigma). Ten milligrams of total protein was pre-cleared on GST-Sepharose (GE Healthcare) prior to incubation with CGIYRLRSC-GST-Sepharose (4 µg peptide/mg total protein) overnight at 4°C. Bound proteins were eluted from Sepharose beads, separated on a 10% SDS–polyacrylamide gel, and stained with BioSafe Coomassie blue (BioRad, Hercules, CA). Specific bands were analysed by mass spectrometry: matrix-assisted laser desorption/ionization (MALDI) mass spectra were recorded on an Applied Biosystems Voyager DE-PRO mass spectrometer equipped with a reflectron time-of-flight (TOF) analyser and used in delayed extraction mode (Applied Biosystems, Foster City, CA). Raw data, reported as monoisotopic masses, were introduced into the MASCOT peptide mass fingerprinting search program (Matrix Science, Boston, MA) for protein identification. Liquid chromatography (LC)-mass spectrometry (MS)/MS analyses were performed on a CHIP MS Ion Trap XCT Ultra equipped with a 1100 high pressure liquid chromatography (HPLC) system and a chip cube (Agilent Technologies, Palo Alto, CA). Analysis was performed by data-dependent acquisition of one MS scan (mass range from 400 to 2000 *m*/*z*) followed by MS/MS scans of the three most abundant ions in each MS scan. Raw data from nanoLC-MS/MS analyses were introduced into the MASCOT software to search the human proteome.

### Preparation of stably transduced U293 cells

FLAG-tagged human angiopoietin-like 6 cDNA, inserted into pcDNA3.1(+).Neo vector (pcDNA3.ANGL6), was a gift from Dr. Y. Oike (Japan Science and Technology Agency, Japan; Oike et al, 2005); human E-cadherin cDNA, cloned into pcDNA3.1(+).Neo vector (pcDNA3.CAD1) was a gift from Dr. C. Gottardi (North Western University Medical School, Chicago, IL; Gottardi et al, 2001); β_4_ integrin cDNA, cloned into PRK5 plasmid (pRK5.ITB4), was purchased from Addgene (Cambridge, MA); human α_6A_ integrin cDNA, inserted into pLXSN plasmid (pLα_6_SN; Tamura et al, 1990), was a gift from Dr. A. Magrelli (La Sapienza University, Rome, Italy). The latter cDNA insert was PCR-amplified with the following primer pair:

5′-AAACTTAAGCTTGCCACCATGGCCGCCGCCGGGCAG-3′ and
5′-TACACGGGCCCTCTATGCATCAGAAGTAAGCCT-3′

and subcloned into pcDNA3.1(+). Hygro vector between *Hind*III and *Apa*I sites to obtain the pcDNA3.ITA6A plasmid. To achieve correct processing and high amounts of α_6A_ integrin, a concomitant expression of its molecular partner β_4_ integrin was necessary. For stable overexpression of α_6A_β_4_ (abbreviated as α_6_ in the text) integrin, E-cadherin, and angiopoietin-like 6, U293 cells were transduced with the described plasmids by the use of a calcium phosphate transfection kit (Invitrogen), followed by selection of single cell clones in culture media supplemented by 500 µg/ml, geneticin (Sigma) and/or 200 µg/ml hygromycin (Invitrogen). Angiopoietin-like 6 was quantified in cell supernatants by using the #96-well ANGPLT6 ELISA Kit (Adipogen, San Diego, CA).

### Preparation of cell lines with silenced expression of target genes

A transient gene silencing approach was applied to cells used for short-term *in vitro* experiments. NCI-H630 cells were transduced with ON-TARGETplus SmartPOOL siRNA for *ITGA6* and *CDH1*, or with control siRNA (Dharmacon, Lafayette, CO). For each experimental point, 2 × 10^5^ cells were transfected with either control, *ITGA6*, *CDH1* or both siRNA pools, according to the manufacturer's protocol. To quantify gene down-modulation, we evaluated RNA and protein levels after 24 and 72 h, respectively. A stable silencing approach was preferred for cells used for long-term, *in vivo* experiments. For this purpose, 2 × 10^5^ HCT-116m, SW-48, HT-29 or DLD-1 cells were transfected with shRNA plasmid pools targeting *ITGA6* or *CDH1*, or with non-targeting control shRNA plasmid pool A (all from Santa Cruz Biotechnologies), according to the manufacturer's protocol. Following selection in medium supplemented with 2.5 µg/ml puromycin (Sigma), six clones for each experimental point were subjected to dotblot analysis to confirm selective protein down-regulation. For this purpose, cell lysates (1 µg each) were spotted onto polyvinylidene fluoride (PVDF) membranes; after drying, membranes were subjected to specific antibody staining with standard procedures.

### Retrotranscription and real-time PCR

RNA was retrotranscribed by the use of the High Capacity cDNA Reverse Transcription Kit (Applied Biosystems) and amplified with the Power SYBR Green PCR Master Mix (Applied Biosystems). For residual transcript quantification in silenced cells, the following primer pairs were used for real-time PCR amplification of the cDNAs in an ABI PRISM 7700 instrument:

*ITGA6*: 5′-TGAGTGTCCCCCGGTATCTTC-3′ and 5′-CAGTATCAGCCGCTTTCAGATTTT-3′;
*CDH1*: 5′-GCTGGTTATAATCCTTCAATATCAATTGT-3′ and 5′-TTGGGCTCAGAACCTTGGTTT-3′;
*GAPDH*: 5′-GAAGGTGAAGGTCGGAGTC-3′ and 5′-GAAGATGGTGATGGGATTTC-3′.

### Immunostaining

OCT-frozen tissues were cut into 10-µm sections, and paraffin-embedded tissues into 5-µm sections. For immunostaining of cell lines, 10^4^ cells were plated on a SuperFrost Plus glass slide (Menzel-Gläser, Braunschweig, Germany) and were grown for 24 h followed by fixation in 4% paraformaldehyde in PBS for 5 min at RT. Immunostaining was performed according to standard protocols. Visible images were acquired with either an EC3 Leica (frozen tissues) or a High-Performance IEEE 1394 FireWire Digital CCD Camera (QIMAGING, Surrey, BC, Canada; paraffin-embedded tissues). Fluorescent images were acquired with either a DMIRE2 confocal microscope or a DMI 3000D microscope equipped with a DFC 360FX digital camera (all from Leica). For quantification of fluorescent signal colocalizations, three images/sample (1024 × 1024 pixels = 375 × 375 µm^2^) were divided in 8-bit images corresponding to the red and green fluorescence channels. Image pairs were analysed with the Image Processing and Analysis software in Java (ImageJ), with the Colocalization Highlighter plugin to create a binary representation of colocalized pixels, and the Image Calculator option to derive the non-colocalized pixels.

### Immunoblot and IP

Cells were lysed in four pack cell volumes of phosphate-buffered saline (PBS), 1 mM CaCl_2_, 1 mM MgCl_2_, 1 mM PMSF, protease inhibitor cocktail, supplemented with either 50 mM β-octyl-d-glucosylpyranoside (interaction studies) or 0.1% NP-40 (expression studies). Tissues were homogenized in a Potter-Elvehjem grinder in the same buffer (∼1 ml/100 mg tissue). Homogenates were cleared by centrifugation and filtration through 0.45 µm pore filters. For IP, lysates were precleared for 1 h at 4°C on Protein G-Sepharose (GE Healthcare), followed by incubation with specific antibodies for 1 h at 4°C and addition of Protein G-Sepharose for 2 h at 4°C. Proteins were separated on 10% SDS–PAGE and were blotted onto PVDF membranes (Millipore, Billerica, MA). For protein quantification, densitometric analysis of the detected bands was performed with QuantityOne software (BioRad); values were normalized to the intensity of vinculin bands.

### *In vitro* assays

To evaluate the capability of soluble angiopoietin-like 6 to bind to adhered cells expressing α_6_ integrin and E-cadherin, mock- or receptor-transduced U293 cells were plated on SuperFrost Plus glass slides (Menzel-Gläser) and were grown for 24 h. Cells were then incubated with 100 µl of conditioned media from angiopoietin-like 6-secreting or mock-transfected U293 cells for 2 h at 4°C, washed three times with PBS, fixed with 4% paraformaldehyde, and stained with the anti-angiopoietin-like 6 rabbit polyclonal antibody using standard protocols. The intensities of fluorescent pixels were quantified in at least 25 ROIs/sample (area = 100 µm^2^), by using the Leica Application Suite software, and reported as percent variation compared to the negative controls. For investigation of cell adhesion to angiopoietin-like 6, 1 µg of the recombinant protein was incubated *per* well of a 96-well plate for 1 h at 37°C. After a blocking step in IMDM, 2% FCS for 1 h at 37°C, 10^4^ cells were allowed to adhere for 1 h at 37°C. Samples were washed in PBS, and cells were fixed in 8% glutaraldehyde and stained in 0.25% crystal violet in 10% methanol. For tissue adhesion, 10-µm sections of OCT-frozen grossly normal liver samples from metastatic CRC patients were blocked in IMDM, 2% FCS for 30 min at 37°C, followed by incubation with 5 × 10^4^ cells in 5% CO_2_ at 37°C for the indicated periods of time. Samples were washed four times in the same medium and once in PBS, fixed in 4% paraformaldheyde, and stained with haematoxylin (BioOptica). Adhered cells were counted manually under a light microscope. To test the peptide effects on cell proliferation, we plated 2 × 10^4^ cells *per* well in a 24-well plate, in the presence of either control or CGIYRLRSC peptide (100 µM). At the indicated time points, cells were fixed in glutaraldehyde, stained in crystal violet and solubilized in 10% acetic acid. Cell growth was evaluated by absorbance at 590 nm in a microplate reader (Perkin Elmer, Waltham, MA).

### SPR analyses

A BIAcore X instrument (GE-Healthcare, Milwaukee, WI) was used. To study the interaction between α_6_ integrin and E-cadherin, the amine coupling immobilization procedure was adopted. Briefly, the Fc-his-tagged extracellular domain of E-cadherin (227 nM) was allowed to react with a CM5 sensor chip (GE-Healthcare) activated with a mixture of 0.2 M EDC and 0.5 M NHS, leading to the immobilization of 11,830 Resonance Unit (RU) (corresponding to 0.134 pmoles/mm^2^) of protein. Similar results were obtained for a reference chip coated with Fc-his-tag-VEGFR2. Sensor chips were deactivated by injection of HCl–ethanolamine. The his-tagged extracellular domain of α_6_β_4_ integrin (100 nM) was injected over the E-cadherin surface for 4 min in 10 mM Hepes pH 7.4 containing 0.15 mM NaCl, 50 µM EDTA, 0.005% Surfactant P20, 1 mM CaCl_2_, 1 mM MgCl_2_ and 1 mM MnCl_2_ (running buffer), and then washed until dissociation. Because the sensor chip could not be properly regenerated, we repeated this analysis on three different, newly prepared E-cadherin-coated sensor chips to estimate a *K*_d_ for the receptor interaction.

To study the interaction of α_6_ integrin and E-cadherin with angiopoietin-like 6, NTA sensor chips (GE-Healthcare) were activated with 500 µM nickel solution, and either his-tagged protein was injected over the activated surfaces to immobilize properly oriented receptors. This procedure led to the immobilization of 1930 and 2000 RU (10.2 and 22.5 fmoles/mm^2^) of protein for α_6_ integrin and E-cadherin, respectively. A sensor chip saturated with nickel was used as a reference. Angiopoietin-like 6 was injected over either sensor chip for 4 min in running buffer, and then washed until dissociation. Following a procedure optimized for SPR analysis on decaying surfaces (Joss et al, 1998), after each injection the sensor chips were subjected to a new immobilization to obtain surfaces with unvarying receptor density. *K*_d_ values were calculated from the overlay of sensorgrams generated by injection of increasing concentrations of angiopoietin-like 6, using the nonlinear fitting (single site model) software package BIAevaluation 3.2 with a drifting baseline. Only sensorgrams whose fitting values of ×2 were <10 were considered in the analysis (Khalifa et al, 2001).

### Animal models

Experiments involving animals were reviewed and approved by the Institute's ethical committee, and by the Italian Ministry of Health. Six-week female CD1-nude mice were purchased from Charles River (Lecco, Italy). Animals were subjected to intraperitoneal anaesthesia with a mixture of 0.75 mg/ml xylazine (Xilor®, BIO98, Milan, Italy), 1 mg/ml tiletamine + 1mg/ml zolazepam (Zoletil®, Virbac, Milan, Italy), in physiological solution. After the mice were deeply anaesthetized, a midline incision was performed and target organs were gently exposed. Two or five million suspended cells were injected in 50 µl of culture medium intrasplenically (Giavazzi et al, 1986) or into the median liver lobe (Kuo et al, 1995), respectively. To investigate a pharmacological intervention on liver homing and/or colonization of CRC cells, we randomized animals in two groups that received medium alone (vehicle) or supplemented with 100 µM CGIYRLRSC peptide. The wound was closed by a double suture, and each animal was given 0.1 mg caprofen (Rymadil®, Pfizer, Milan, Italy) in a physiological solution to allow post-operative pain relief and rehydration. Mice were strictly monitored until completely awake, and oral ampicillin was administered for 5 days after surgery. Mice were euthanized at the indicated time points, and organs were photographed with a PL-200 digital photocamera (Samsung Electronics, Milan, Italy). External metastatic areas were quantified by the use of ImageJ software.

**The paper explained**

PROBLEM:

CRC remains a leading cause of cancer death, mainly due to metastatic spreading. New approaches are therefore urgently needed to improve the management of patients with advanced CRC.

RESULTS:

We here describe a molecular mechanism by which a physical and functional interaction between a novel ligand/receptor pair (angiopoietin-like 6 and α_6_ integrin/E-cadherin) contributes to liver homing and colonization of human CRC cells. We also describe an angiopoietin-like 6-mimicking peptide capable of interfering with this interaction, thus acting as an antimetastatic compound. We finally provide evidence for a correlation between high levels of these molecules and poor prognosis in patients with metastatic CRC.

IMPACT:

The identified metastasis binding, angiopoietin-like 6-mimicking peptide can be the basis for the development of an antimetastatic drug(s) with the potential to be translated into a clinical trial. Furthermore, the coexpression of α_6_ integrin and E-cadherin in CRC tissues can be exploited as a new prognostic marker that could support physicians in evaluating diagnostic and/or therapeutic interventions.

### Bioinformatics

The frequency of peptide motifs was evaluated with ClustalW. The protein Basic Local Alignment Search Tool (BLAST) was used to investigate similarities between the metastasis-binding peptides and the human proteome. To extract only the extracellular and transmembrane proteins from the BLAST output dataset, Gene Ontology_Cell Component (GO_CC) annotations were retrieved through the DAVID Bioinformatics Resource Functional Annotation tool (Huang da et al, 2009), searching for GOTERM_CC_ALL, with default settings.

### Statistical analyses

All the analyses were performed with Prism 5 software (GraphPad, La Jolla, CA): two-way analysis of variance (ANOVA) followed by Bonferroni's post-test was used to evaluate differences within treatments; *t*-test and Fisher's exact test (two-tailed) were used to compare selected experimental points; the Chi-squared test was used to analyse contingency tables; survival curves were drawn as Kaplan–Meier Cumulative Proportion Surviving graphs, and corresponding *p*-values were calculated by the use of the log-rank (Mantel-Cox) test. Asterisks indicate the following *p*-value ranges: *p < 0.05, **p < 0.01, ***p < 0.001.

For more detailed Materials and Methods see the Supporting Information.

## Author contributions

SM conceived the project, performed the phage display biopanning and part of the *in vitro* experiments, wrote the manuscript, prepared the figures, and provided research founding; MS performed *in vivo* experiments and contributed to the preparation of the figures; SC performed *in vivo* experiments; AM managed the collection of human samples and performed the analysis of clinical data; ABa performed the immunostaining of mouse tissues and contributed to the preparation of the figures; VB performed *in vivo* and *in vitro* experiments; DR performed *in vivo* experiments; MM performed the proteomics analyses; PB performed *in vitro* experiments; JS performed the bioinformatics analyses; RG supported the set up of the HCCM-1544 model; SA and PC managed the collection of human samples; LC supported the collection of human samples and provided a critical evaluation of the manuscript; PP supported the proteomics analyses and provided research founding; ABu and MR performed the SPR analyses and contributed to the revision of the manuscript; RP and WA coordinated the phage display and bioinformatics approaches, contributed to the preparation of the manuscript, and provided research founding; FB contributed to the preparation of the manuscript and provided research founding.

## Supplementary material

Detailed facts of importance to specialist readers are published as ”Supporting Information”. Such documents are peer-reviewed, but not copy-edited or typeset. They are made available as submitted by the authors.
