# Supplementary material for: A complex of α6 integrin and E-cadherin drives liver metastasis of colorectal cancer cells through hepatic angiopoietin-like 6
Source: EMBO Mol Med. 2012 Oct 16;4(11):1156–75. doi: 10.1002/emmm.201101164 (PMC3494873; doi:10.1002/emmm.201101164)
Supplement: Supplementary file 1 [file emmm0004-1156-SD1.pdf]

EMBO
Molecular Medicine

Manuscript EMM-2011-01164

A complex of 6 integrin and E-cadherin drives liver metastasis of colorectal cancer cells through hepatic angiopoietin-like 6

Serena Marchiò, Marco Soster, Sabrina Cardaci, Andrea Muratore, Alice Bartolini, Vanessa Barone, Dario Ribero, Maria Monti, Paola Bovino, Jessica Sun, Raffaella Giavazzi, Sofia Asioli, Paola Cassoni, Lorenzo Capussotti, Piero Pucci, Antonella Bugatti, Marco Rusnati, Renata Pasqualini, Wadih Arap, Federico Bussolino

Corresponding author: Serena Marchio, Institute for Cancer Research and Treatment

Review timeline:

Submission date:	20 December 2011
Editorial Decision:	20 February 2012
Revision received:	15 June 2012
Editorial Decision:	23 July 2012
Revision received:	04 September 2012
Accepted:	07 September 2012

Transaction Report:

(Note: With the exception of the correction of typographical or spelling errors that could be a source of ambiguity, letters and reports are not edited. The original formatting of letters and referee reports may not be reflected in this compilation.)

1st Editorial Decision 20 February 2012

Thank you for the submission of your manuscript to EMBO Molecular Medicine and please accept my sincere apologies for the long delay in getting back to you. We have now heard back from the referees who were asked to evaluate your manuscript.

As you will see from the enclosed reports, they both find the topic of your manuscript potentially important. However, while Referee #2 mainly recommends some clarification and further discussion, Referee #1 recommends performing extra experiments that this reviewer feels are necessary for the paper to be conclusive.

Given these evaluations, I would like to give you the opportunity to revise your manuscript, with the understanding that the referee concerns must be fully addressed and that acceptance of the manuscript would entail a second round of review. Please note that it is EMBO Molecular Medicine policy to allow only a single round of revision and that, as acceptance or rejection of the manuscript will depend on another round of review, your responses should be as complete as possible.

Revised manuscripts should be submitted within three months of a request for revision; they will

otherwise be treated as new submissions, except under exceptional circumstances in which a short extension is obtained from the editor. Also, the length of the revised manuscript may not exceed 60,000 characters (including spaces) and, including figures, the paper must ultimately fit onto optimally ten pages of the journal. You may consider including any peripheral data (but not methods in their entirety) in the form of Supplementary information.

I look forward to seeing a revised form of your manuscript as soon as possible.

Yours sincerely,

Editor
EMBO Molecular Medicine

***** Reviewer's comments *****

Referee #1:

This work shows that newly identified peptides mimicking angiopoietin-like 6 inhibited the interaction between hepatic angiopoietin-like 6 and tumor 6 integrin/E-cadherin and thus metastasis of colorectal cancer cells. This is potentially very important.

I have one major concern. The biochemistry of the angiopoietin-like 6/ 6 integrin/E-cadherin complex is only poorly characterized. Supernatants probably containing angiopoietin-like 6 are used. Instead advanced biochemical characterization by proteomics, using all recombinant proteins, is required. These techniques are available in Candiolo. Interesting is also whether integrin 6- and E-cadherin interact with each other on the outside or inside of cells, and whether angiopoietin-like 6 binds to one or both cell adhesion molecules. Without these data, the results of the paper remain doubtful.

Referee #2 (Comments on Novelty/Model System):

This is a novel, potentially important study that could shed light on possible mechanisms that control organ-specific tumor cell metastasis. The authors appear to have conducted careful and thoughtful experiments and the paper appears to be well-written.

Referee #2 (Other Remarks):

Manuscript #EMM-2011-01164 "A complex of alpha6 integrin and E-cadherin drives the liver metastasis of colorectal cancer cells by a physical and functional interaction with hepatic angiopoietin-like 6" by Serena Marchio et al. describes a novel, potentially important study that could shed light on possible mechanisms that control organ-specific tumor cell metastasis. The authors appear to have conducted careful and thoughtful experiments and the paper appears to be well-written.

This reviewer recommends publication of this paper pending the answering of some specific concerns:

1. The authors have apparently made some assumptions about their putative target proteins. They appear to have ignored membrane-bound factors in their BLAST search for proteins with the GIYRLRS and GVYSLRS sequences and they have only considered results only for adhesion proteins in Table 1S. Presumably the rationale is based on previously published work. This work needs to be discussed in the Discussion or Introduction.

2. It would be very useful to see what other proteins came up in the BLAST search in order to compare angiopoietin-like 6 with other candidates.

3. The angiopoietin-like 6-mimicking peptides and fusion proteins contain Cys residues. Disulfide bond formation could result in either cyclized peptides or formation of dimmers and even higher multimers. How was disulfide bond formation prevented? If no precautions were taken to prevent disulfide bond formation, what percentage of the angiopoietin-like 6-mimicking peptides was in the form of monomers, cyclized peptides, and higher multimers? When these peptides were used in adhesion, proliferation, and in vitro assays, how do the authors exclude the possibility that these peptides form disulfide bonds with other proteins on the cell surface or in the extracellular milieu and it is these proteins crosslinked to the peptides that are actually the bioactive component?

4. The authors need to reconcile their results with those of Kim et al (Biochem J 346:603-610) indicating that angiopoietin-like 6 is only found in the liver. The observations in figure 2 suggest that angiopoietin-like 6 is found in organs other than the liver. In the in vivo experiments, do the authors observe any metastasis of CRC cells to those organs also? If not, what are the possible reasons and how does this change the conclusions of the paper?

5. In figure 5, the authors used cells in which expression of integrin alpha6 and E-cadherin were knocked down. The authors should consider using stably transfected cells in experimental metastasis experiments showing that alpha6 integrin and E-cadherin are both required for experimental metastasis to the liver. Alternatively, inhibitory anti-integrin alpha6 and E-cadherin antibodies could be used in the experimental metastasis assays.

1st Revision - Authors' Response 15 June 2012

Referee #1 (Other Remarks):

This work shows that newly identified peptides mimicking angiopoietin-like 6 inhibited the interaction between hepatic angiopoietin-like 6 and tumor α6 integrin/E-cadherin and thus metastasis of colorectal cancer cells. This is potentially very important.

I have one major concern. The biochemistry of the angiopoietin-like 6/α6 integrin/E-cadherin complex is only poorly characterized. Supernatants probably containing angiopoietin-like 6 are used. Instead advanced biochemical characterization by proteomics, using all recombinant proteins, is required. These techniques are available in Candiolo. Interesting is also whether integrin α6 and E-cadherin interact with each other on the outside or inside of cells, and whether angiopoietin-like 6 binds to one or both cell adhesion molecules. Without these data, the results of the paper remain doubtful.

For the assays involving angiopoietin-like 6-containing supernatants, an ELISA quantification of the protein amounts has now been included.
The biochemical characterization of the described molecular interactions required a long and complex set up, which is here partially reported. These analyses were finally successful and demonstrated that (i) integrin α6 and E-cadherin interact with each other through their extracellular domains, therefore on the outside of the cells; (ii) angiopoietin-like 6 binds both adhesion molecules with similar affinity.
To characterize the interactions of the recombinant proteins, we used a BIAcore X instrument, with different experimental approaches (results are now included, page 7 line 11 and page 8 line 16, and are illustrated in Fig 3D and Fig 4E):
(i) Receptor/ligand interaction. Both E-cadherin and integrin α6β4 (recombinant extracellular portions) have a His-tag that was exploited to obtain surfaces with properly oriented receptors. Different concentrations of angiopoietin-like 6 were injected over the E-cadherin or integrin α6β4 surfaces; unfortunately, after ligand binding and subsequent regeneration, part of the immobilized receptors dissociated from the surface. We therefore moved to a different procedure, described in the literature, involving a new receptor immobilization after each injection of the ligand (to replenish the surface and to allow analyses on a surface containing an unvarying density of receptors). This protocol modification allowed proper measurements of the dissociation constant (Kd) values, which were 2.03 nM and 29.0 nM for the interaction of angiopoietin-like 6 with E-cadherin and with integrin α6β4, respectively.

ii) Receptor/receptor interaction. This could not be analyzed with the above described assay: because both proteins are His-tagged, the interaction with the chip surface would predominate over protein-protein binding. For this reason, we adopted an amine coupling immobilization procedure to obtain an E-cadherin-coated surface. Unfortunately, after integrin α6β4 injection, the sensor chip could not be regenerated properly, hampering the performing of dose-response experiments on the same sensor chip as required to calculate kinetic parameters with a standard approach. To substantiate the E-cadherin/integrin α6β4interaction, we repeated single injections of the integrin on different, newly prepared E-cadherin-coated sensor chips, obtaining Kd values from 5.3 to 16.8 nM.

Referee #2:

Manuscript #EMM-2011-01164 "A complex of alpha6 integrin and E-cadherin drives the liver metastasis of colorectal cancer cells by a physical and functional interaction with hepatic angiopoietin-like 6" by Serena Marchio et al. describes a novel, potentially important study that could shed light on possible mechanisms that control organ-specific tumor cell metastasis. The authors appear to have conducted careful and thoughtful experiments and the paper appears to be well written.

This reviewer recommends publication of this paper pending the answering of some specific concerns:

1. The authors have apparently made some assumptions about their putative target proteins. They appeared to have ignored membrane-bound factors in their BLAST search for proteins with the GIYRLRS and GVYSLRS sequences and they have only considered results only for adhesion proteins in Table 1S. Presumably the rationale is based on previously published work. This work needs to be discussed in the Discussion or Introduction.

We exploited the specificity of the phage display-selected, metastasis binding peptides in combined biochemical/bioinformatics approaches to characterize the interacting partners both from the ligand (peptide-like) and from the receptor (peptide-binding) sides. This approach is quite complicate, and we apologize if our report was confusing: we here attempt to better clarify our experimental plan and results. In particular, the choice of potential candidates was not based on previously published work, and is here briefly explained.
Ligand side. We performed a BLAST analysis to explore the human proteome in search for proteins similar to the two metastasis-binding peptides GIYRLRS and GVYSLRS: we considered as true positive matches only the transmembrane and extracellular proteins (i.e. proteins that can bind the potential receptors from the external side of the cells). Angiopoietin-like 6 appeared to be a good candidate, because it shares sequence similarity with the peptides in two regions of its fibrinogen domain (the output of the cited BLAST analysis has now been added as table S1).

Receptor side. To isolate putative targets of the GIYRLRS sequence, a corresponding peptide was produced and purified as GST-fused protein, and this bait was used to "fish" candidate receptors present on the surface of metastatic cells. Proteins extracted from NCI-H630 cells (hepatic metastasis from CRC), and specifically pulled-down by CGIYRLRSC-GST + Glutathione-Sepharose, were identified by mass-spectrometry. In Table S1 (now Table S2), all the proteins that reached a MASCOT identification score ≥ 50 were listed. Because the phage display was performed on intact cells, the receptor(s) for the selected metastasis-binding peptides should be either transmembrane or secreted proteins. Therefore, cytoplasmic, mitochondrial, or nuclear proteins that have been identified by mass spec are either (i) false positive, or (ii) part of a transducing unit starting from the cell surface (transmembrane protein) and signaling inside the cell. The latter is true, for example, if one considers an adhesion protein with associated cytoskeletal proteins. For all these reasons, we chose α6 integrin and E-cadherin, i.e., the adhesion proteins that obtained the highest identification score, and we investigated these two candidates as potential receptors for the metastasis-specific peptides (and for angiopoietin-like 6).

2. It would be very useful to see what other proteins came up in the BLAST search in order to compare angiopoietin-like 6 with other candidates.

Some of the protein mimicked by our metastasis-binding peptides were cited in the discussion; in the revised version of the manuscript, a table listing the result of the BLAST analysis is now included as Table S1.

3. The angiopoietin-like 6-mimicking peptides and fusion proteins contain Cys residues. Disulfide bond formation could result in either cyclized peptides or formation of dimmers and even higher multimers. How was disulfide bond formation prevented? If no precautions were taken to prevent disulfide bond formation, what percentage of the angiopoietin-like 6-mimicking peptides was in the form of monomers, cyclized peptides, and higher multimers? When these peptides were used in adhesion, proliferation, and in vitro assays, how do the authors exclude the possibility that these peptides form disulfide bonds with other proteins on the cell surface or in the extracellular milieu and it is these proteins cross-linked to the peptides that are actually the bioactive component?

Peptides were produced and purified as 100% linear (confirmed by their mass-spec analysis provided by New England Peptide, Inc), stocked frozen as a lyophilized powder, and solubilized in water immediately before their use; therefore they were added in their linear version in all the described assays. However, the formation of disulfide bonds was not prevented; all the experiments involving the use of synthetic peptides were performed in physiological conditions, i.e. in culture medium without the addition of any reducing agent.
It is therefore likely that dimmers/multimers form during time. This does not change the peptide capability to recognize its target: for another project, focused on cancer imaging, we have functionalized fluorescent silica nanoparticles with either monomeric (with only the N-terminal Cys) or dimeric (both Cys and forced dimerization) GIYRLRS or GVYSLRS peptides, and the corresponding nanocarriers target equally well the hepatic metastases.

However, a problem of peptide availability due to its sequestering by serum proteins might be relevant for in vivo applications. This is the reason why we do not treat tumor-bearing mice i.v. with the peptides; instead, we mix tumor cells and peptides immediately before their surgical implantation. A molecular evolution of our peptides (PEGylation, dendrimers, coupling to nanoparticles...) for future clinical applications is for sure needed, and this will be a possible industrial development for metastasis-targeted diagnostic/therapeutic tools.

4. The authors need to reconcile their results with those of Kim et al (Biochem J 346:603-610) indicating that angiopoietin-like 6 is only found in the liver. The observations in figure 2 suggest that angiopoietin-like 6 is found in organs other than the liver. In the in vivo experiments, do the authors observe any metastasis of CRC cells to those organs also? If not, what are the possible reasons and how does this change the conclusions of the paper?

Kim et al. found angiopoietin-like 6 mRNA exclusively in the liver in humans. We therefore assumed, at first, that also the corresponding protein was present in the liver only; however, our data showed that it is detectable (although in lower amounts) in various organs (Figure 2).

From a technical point of view, IHC analyses of protein expression are much more sensitive than Northern Blot evaluation of mRNA: if only a subset of cells in a tissue express the mRNA, or if the whole tissue expresses it in low amounts, its presence might not be detected. On the contrary, IHC allows the detection of even a single positive cell surrounded by negative tissue. A statement on this issue has been added in the revised manuscript (page 6 line 2). From a biological point of view, it is also possible that at least part of the angiopoietin-like 6 observed in distant organs (especially in blood vessels) might have reached these organs through the bloodstream. A release of angiopoietin-like 6 from the liver and its consequent presence into the bloodstream has been described in physiological and pathological conditions (Boztosun et al. J Investig Med. 2012; Okazaki et al. B. J Dermatol. 2012; Namkung et al. Metabolism. 2011; Ebert et al. Metabolism. 2009; Stepan et al. Am J Hypertens. 2009). Besides our IHC staining, the presence of angiopoietin-like 6 is confirmed by a large-scale analysis performed on normal and tumor human tissues and reported in Protein Atlas (http://www.proteinatlas.org/ENSG00000130812).

Among the organs in which angiopoietin-like 6 is present, the lungs are of interest because they represent a typical metastatic destination of CRC cells. A statement on this issue has been added in the revised manuscript (page 6 line 3). We observed high amounts of angiopoietin-like 6 in normal lungs (Figure 2), and high levels of α6 integrin and E-cadherin colocalization in lung metastases from CRC (Figure 9). These data are consistent with our molecular model for the metastatic addressing of cancer cells. Unfortunately, in our mouse models we did not observe metastatic foci in the lungs, so a proper animal model (different cell lines/tissues or cell inoculation site) should be

developed to deeply investigate this very interesting issue.

5. In figure 5, the authors used cells in which expression of integrin alpha6 and E-cadherin were knocked down. The authors should consider using stably transfected cells in experimental metastasis experiments showing that integrin alpha6 and E-cadherin are both required for experimental metastasis to the liver. Alternatively, inhibitory anti-integrin alpha6 and E-cadherin antibodies could be used in the experimental metastasis assays.

From the gain-of-function point of view: we have U293 cells stably overexpressing integrin alpha6, E-cadherin, or both, which were used for the in vitro assays; however, these cells are not from CRC, and they are not a good system for in vivo applications. All the CRC lines tested express integrin alpha6 and E-cadherin in medium-to-high amounts (Figure S2), and all of them are metastatic. Interesting, HCT-116 express barely detectable levels of these proteins: despite being morphologically and biologically (proliferation, basal motility) comparable to HCT-116m, they do not metastasize to the liver. From the loss-of-function point of view: from the very beginning of this project we attempted to produce cell clones stably silenced for both of integrin alpha6 and E-cadherin, but we could not obtain an acceptable level of concomitant down regulation. This is possibly due to a cross-regulation of the respective gene expression, or to the fact that the presence of at least one of these proteins is indispensable for cell viability. For this reason, we performed the in vivo experiments with single-silenced cells; however, we demonstrated that even an incomplete depletion of only a receptor protein is sufficient to alter liver colonization by CRC cells. However, we do agree with Referee #2 that both approaches need to be implemented; they are part of our future planning.

The use of blocking antibodies requires a separate discussion. Because the interaction of integrin alpha6 with E-cadherin and of both with angiopoietin-like 6 is non-canonical, our opinion is that blocking (i) integrin alpha 6laminin interaction and cell-matrix contacts (for example, with the rat monoclonal GoH3) or (ii) E-cadherin/E-cadherin interaction and cell-cell contacts (with the rat monoclonal DECMA-1) might confuse the results. Conversely, we are planning to develop specific antibodies that can block integrin alpha6/E-cadherin, integrin alpha6/angiopoietin-like 6, and/or E-cadherin/angiopoietin like 6 interactions. In this revised version of the manuscript we provide a deep molecular characterization of such interactions (results are now included, page 7 line 11 and page 8 line 16, and are illustrated in Fig 3D and Fig 4E), which will be the basis for rationally designed neutralizing antibodies.

2nd Editorial Decision 23 July 2012

Thank you for the submission of your revised manuscript to EMBO Molecular Medicine. We have now received the enclosed reports from the referees that were asked to re-assess it. As you will see the reviewers are now supportive and I am pleased to inform you that we will be able to accept your manuscript pending the following editorial final amendments:

- provide up to 5 keywords
- provide an ethical statement regarding the use of animals within the Material and Methods section
- accept all changes form the mansucript text
- organise figures 1, 4 and 9 in a portrait format and increase the labels. As these figures are quite large they will have to be reduced and the labels will likely not be readable any longer.
- add the corresponding page numbers to the Table of Content of the Supplementary Information file.

Please submit your revised manuscript within two weeks. I look forward to seeing a revised form of your manuscript as soon as possible.

Yours sincerely,

Editor
EMBO Molecular Medicine

***** Reviewer's comments *****

Referee #1:

I'm satisfied with the revision made.

Referee #2:

This revised version of manuscript #2012-07-03 by Marchio et al is greatly improved over the original version and is now suitable for publication in EMBO Molecular Medicine.

2nd Revision - Authors' Response 04 September 2012

(The authors have amended the manuscript regarding all editorial requirements)

© EMBO
