# Supplementary material for: A complex of α6 integrin and E-cadherin drives liver metastasis of colorectal cancer cells through hepatic angiopoietin-like 6
Source: EMBO Mol Med. 2012 Oct 16;4(11):1156–75. doi: 10.1002/emmm.201101164 (PMC3494873; doi:10.1002/emmm.201101164)
Supplement: Supplementary file 2 [file emmm0004-1156-SD2.pdf]

Supplementary Information

A complex of α_6 integrin and E-cadherin drives the liver metastasis of colorectal cancer cells by a physical and functional interaction with hepatic angiopoietin-like 6

Serena Marchiò[1,2,3,12], Marco Soster[1,2], Sabrina Cardaci[1,2], Andrea Muratore[4], Alice Bartolini[1,2], Vanessa Barone[1,2], Dario Ribero[5], Maria Monti[6], Paola Bovino[1,2], Jessica Sun[7], Raffaella Giavazzi[8], Sofia Asioli[9], Paola Cassoni[9], Lorenzo Capussotti[4], Piero Pucci[5], Antonella Bugatti[10], Marco Rusnati[10], Renata Pasqualini[7], Wadih Arap[7], Federico Bussolino[3,11]

[1] Dept of Oncological Sciences, University of Turin, Italy; [2] Lab of Tumor Microenvironment, Institute for Cancer Research and Treatment (IRCC), Candiolo, Italy; [3] APAvadis Biotechnologies srl, BioIndustry Park S. Fumero, Colleretto Giacosa, Italy; [4] Unit of Surgical Oncology, IRCC; [5] Unit of Hepato-Biliary-Pancreatic and Digestive Surgery, Mauriziano Hospital, Turin, Italy; [6] CEINGE Advanced Biotechnology, Dept of Organic Chemistry and Biochemistry, Federico II University, Naples, Italy; [7] David H. Koch Center, The University of Texas MD Anderson Cancer Center, Houston TX; [8] Lab of Biology and Treatment of Metastasis, Dept of Oncology, Mario Negri Institute for Pharmacological Research, Milan, Italy; [9] Dept of Biomedical Sciences and Human Oncology, University of Turin, Turin, Italy; [10] Dept of Biomedical Sciences and Biotechnology, University of Brescia, Italy; [11] Lab of Vascular Oncology, IRCC

Contents:

Supplementary Figure 1 (Figure 1S) **Pag. 2**

Supplementary Figure 2 (Figure 2S) **Pag. 3**

Supplementary Figure 3 (Figure 3S) **Pag. 4**

Supplementary Table 1 (Table 1S) **Pag. 5**

Supplementary Table 2 (Table 2S) **Pag. 6**

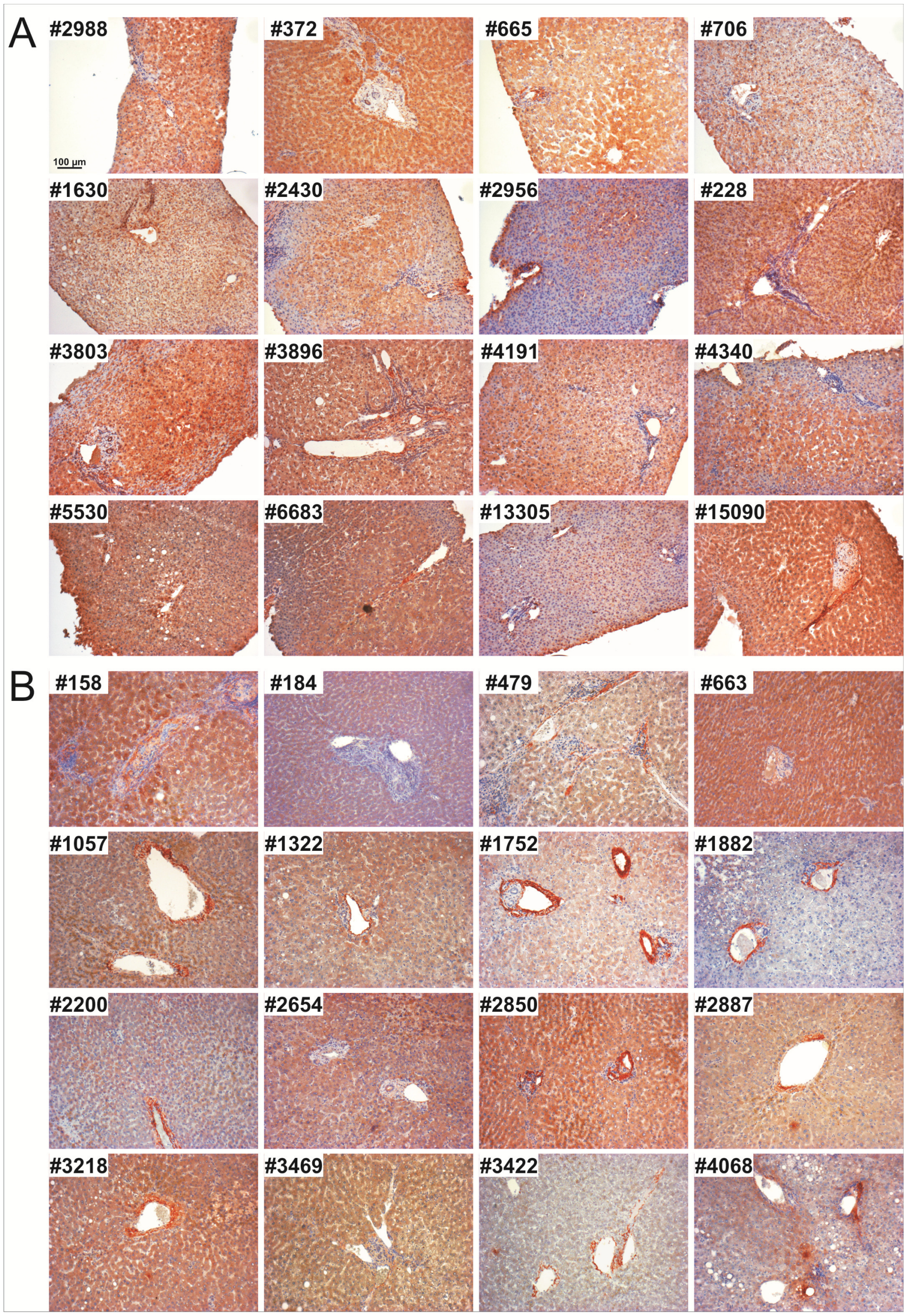

Figure 1S. Angiopoietin-like 6 has a different expression pattern in livers from patients with metastatic CRC compared to livers from healthy donors. A,B. The amounts and localization of angiopoietin-like 6 in livers from healthy donors (n=17) (A) and from patients with metastatic CRC (n=79) (B) were evaluated by staining of 5-µm paraffin-embedded tissue sections. Exemplary pictures of 16 samples for each tissue panel are shown. Numbers refer to the histological archive classification.

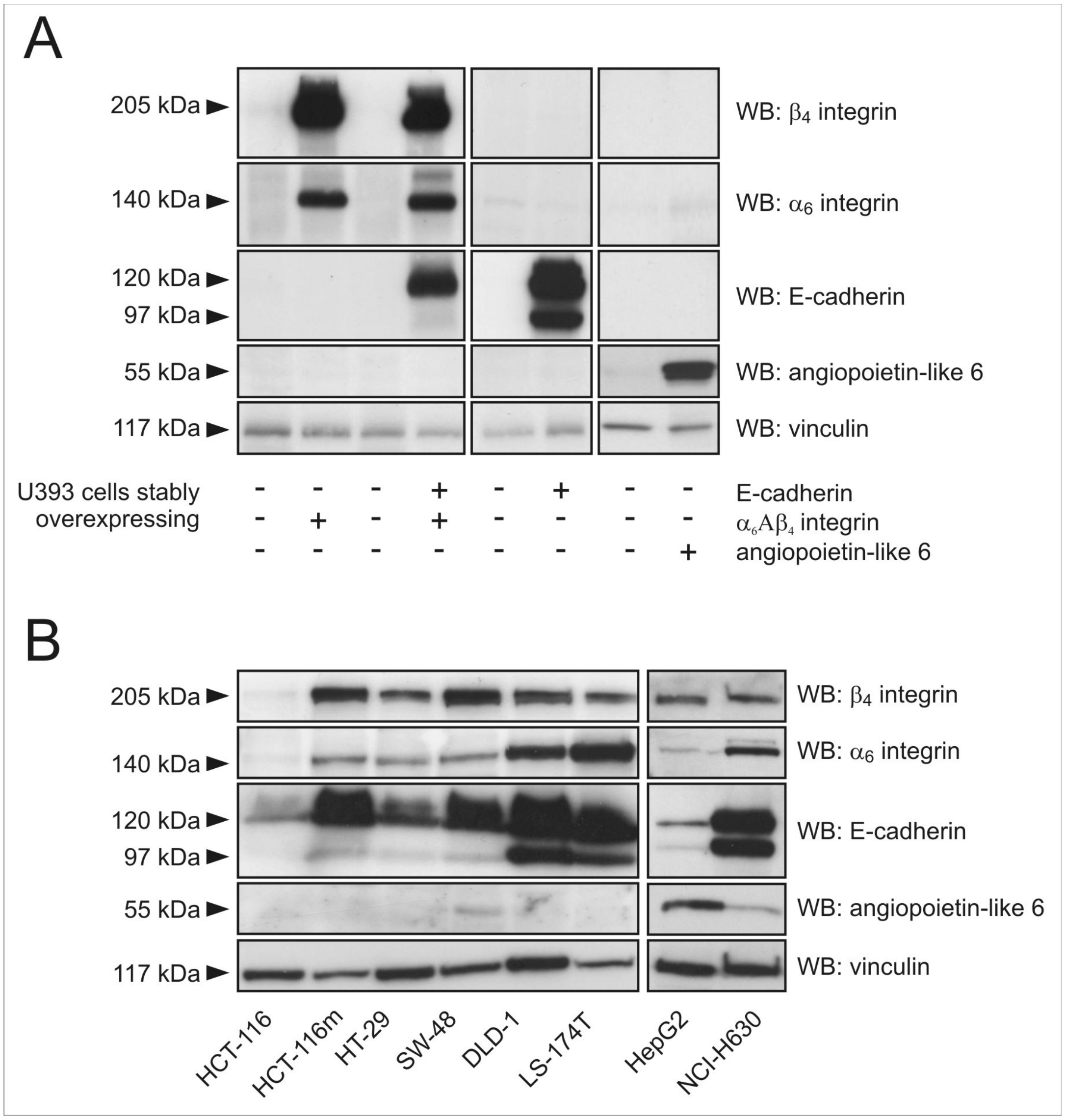

Figure 2S. Protein quantification in all the described cell lines.

A,B. For protein quantification, 50 µg of total lysate was loaded on a 10% SDS-polyacrylamide gel, and proteins resolved by electrophoresis were blotted onto a PVDF membrane. Membranes were stained with the following primary antibodies: mouse monoclonal anti-β_4 integrin clone 7, goat polyclonal anti-α_6 integrin N-19, mouse monoclonal anti-E-cadherin clone 36, mouse monoclonal anti-angiopoietin-like 6 clone Kairos-60, goat polyclonal anti-vinculin N-19. Vinculin was used as a loading control. (A) U293 cells stably over-expressing E-cadherin, $\alpha_{6A}\beta_4$ integrin, a combination of both, or angiopoietin-like 6, (B) CRC cell lines.

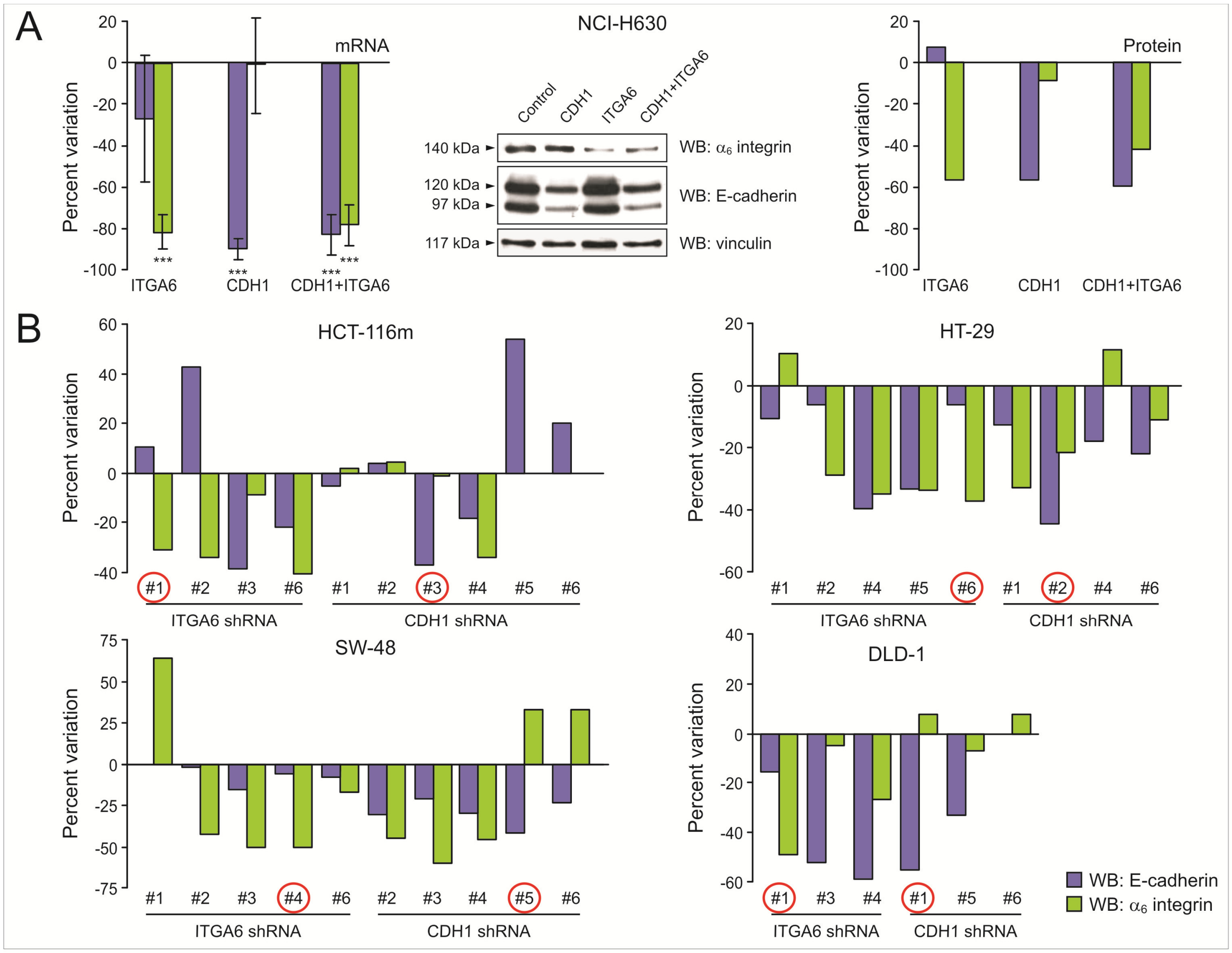

Figure 3S. Validation of α_6 integrin and E-cadherin downmodulation in silenced cell lines. A. Quantification of specific mRNA and protein levels in NCI-H630 cells transiently silenced for the expression of *ITGA6* and *CDH1* mRNAs. Messenger RNA amounts were evaluated after 24 hours by retrotranscription and Real Time PCR amplification of the specific cDNAs. A reduction of 75-85% in both mRNA levels was observed. Protein amounts were evaluated after 72 hours by Western Blot. A reduction of 60-70% in both protein levels was observed. Vinculin staining was used as a loading control. Results are shown as mean ± standard deviation of 9 independent transfections. **B**. Quantification of protein levels in CRC cell lines stably silenced for the expression of E-cadherin and α_6 integrin. HCT-116m, HT-29, SW-48, and DLD-1 cells were transfected with shRNA plasmid pools targeting *ITGA6* or *CDH1*; non-targeting control plasmid pool A was used a s a negative control. Following antibiotic selection, 6 clones each were analyzed by dotblot to confirm specific protein down-regulations. Red circles indicate clones selected for successive experiments, in which a reduction of at least 30-60% in protein levels was achieved. Differences were evaluated for their statistical significance by ANOVA followed by Bonferroni's post-test.

UniProt	Name	Localization	Peptide-mimicked sequence (aa)	Domain
Q8NI99	angiopoietin-like 6	soluble	272-277	Fibrinogen
			454-459	
P24043	laminin alpha 2	soluble	1397-1403	Laminin EGF-like 14
Q9GZU5	nyctalopin	soluble	204-210	LRR6-7
P10451	osteopontin	soluble	164-169	
P98160	perlecan	soluble	3017-3022	Ig-like C2-type 15
Q14392	garpin	transmembrane	441-447	LRR-15-16
Q9UIW2	plexin A1	transmembrane	247-252	Sema
O14917	protocadherin 17	transmembrane	534-539	Cadherin 5
O95206	protocadherin 8	transmembrane	561-566	Cadherin 5
Q9Y5G8	protocadherin γ-A5	transmembrane	163-169	Cadherin 2
Q9BZZ2	sialoadhesin	transmembrane	1391-1397	Ig-like C2-type 14
Q8WWQ8	stabilin-2	transmembrane	2198-2204	Link

Table 1S. GIYRLRS and GVYSRLS mimic adhesion and matrix proteins. A BLAST analysis was performed to investigate possible sequence similarities between the two metastasis-binding peptides (GIYRLRS and GVYSLRS) and the human proteome. This analysis revealed that a number of channel proteins and of seven-pass G-protein coupled receptors share parts of these sequences in common domains; these proteins are therefore not listed in the table. Transmembrane and soluble proteins specifically identified (with score > 25 and e-value < 30) are shown, and their SwissProt IDs, name, localization, peptide-mimicked sequence, and corresponding protein domain are listed.

UniProt	Protein name	Score	Localization	Function
P12830	E-cadherin	173	cell surface	adhesion
P23229	α_6 integrin	98	cell surface	adhesion
P56470	galectin-4	75	cell surface	adhesion
P05026	Na/K-ATPase	94	cell surface	channel
P16444	microsomal dipeptidase	117	cell surface	enzyme
P07900	HSP90	125	cytoplasm	chaperone
P35579	myosin-9	4750	cytoplasm	cytoskeleton
Q7Z406	myosin-14	2408	cytoplasm	cytoskeleton
Q01082	spectrin	1989	cytoplasm	cytoskeleton
O94832	myosin Id	1804	cytoplasm	cytoskeleton
P09327	villin-1	1782	cytoplasm	cytoskeleton
Q00610	clathrin1	1556	cytoplasm	cytoskeleton
O43795	myosin Ib	1252	cytoplasm	cytoskeleton
P07355	annexin A2	994	cytoplasm	cytoskeleton
O00159	myosin Ic	908	cytoplasm	cytoskeleton
P60709	actin	895	cytoplasm	cytoskeleton
Q13813	spectrin	790	cytoplasm	cytoskeleton
Q9NYL9	tropomodulin-3	758	cytoplasm	cytoskeleton
Q12965	myosin Ie	583	cytoplasm	cytoskeleton
P06753	tropomyosin 3	420	cytoplasm	cytoskeleton
P68363	α-tubulin	258	cytoplasm	cytoskeleton
P09525	annexin A4	237	cytoplasm	cytoskeleton
P35580	myosin-10	235	cytoplasm	cytoskeleton
Q9P2M7	cingulin	224	cytoplasm	cytoskeleton
O15143	actin-related protein 2/3 sub1B	193	cytoplasm	cytoskeleton
P35611	α-adducin	98	cytoplasm	cytoskeleton
O15144	actin-related protein 2/3 sub2	96	cytoplasm	cytoskeleton
P68371	β-tubulin	84	cytoplasm	cytoskeleton
Q9UJZ1	stomatin-like protein 2	81	cytoplasm	cytoskeleton
P09874	poly(ADP-ribose) polymerase	106	cytoplasm	enzyme
P16152	NADPH-carbonyl reductase	74	cytoplasm	enzyme
P63092	G-nucleotide-binding protein	98	cytoplasm	G protein
P61247	40S ribosomal protein S3a	224	cytoplasm	ribosome
P45880	voltage-dependent channel	111	mitochondrium	channel
P25705	ATP synthase	200	mitochondrium	enzyme
P19338	nucleolin	236	nucleus	chromatin binding
Q00839	hnRNPU	124	nucleus	DNA/RNA binding

Table 2S. CGIYRLRSC is a candidate ligand for an adhesion complex on hepatic metastasis cells. NCI-H630 (target) and HepG2 (control) cell lysates were incubated with GST-CGIYRLRSC. Selectively bound protein were separated by gel elecrophoresis and were identified by LC-MS/MS. UniProt IDs, protein names and MASCOT identification scores of the identified proteins are listed. General protein localizations/functions are also shown.
